# DNA-PK inhibition enhances neoantigen diversity and increases T cell responses to immunoresistant tumors

**DOI:** 10.1172/JCI180278

**Published:** 2024-10-22

**Authors:** Allison J. Nielsen, Gabriella K. Albert, Amelia Sanchez, Jiangli Chen, Jing Liu, Andres S. Davalos, Degui Geng, Xander Bradeen, Jennifer D. Hintzsche, William Robinson, Martin McCarter, Carol Amato, Richard Tobin, Kasey Couts, Breelyn A. Wilky, Eduardo Davila

**Affiliations:** 1 Department of Medicine, Division of Medical Oncology, University of Colorado School of Medicine, Aurora, Colorado, USA.; 2 Department of Veterans Affairs, Research Service, Rocky Mountain Regional Veterans Affairs, Aurora, Colorado, USA.; 3 PherDal Science, Dixon, Illinois, USA.; 4 University of Colorado Comprehensive Cancer Center and; 5 Department of Surgery, University of Colorado School of Medicine, Aurora, Colorado, USA.

**Keywords:** Immunology, Oncology, Adaptive immunity, Antigen presentation, Cancer immunotherapy

## Abstract

Effective antitumor T cell activity relies on the expression and MHC presentation of tumor neoantigens. Tumor cells can evade T cell detection by silencing the transcription of antigens or by altering MHC machinery, resulting in inadequate neoantigen-specific T cell activation. We identified the DNA–protein kinase inhibitor (DNA-PKi) NU7441 as a promising immunomodulator that reduced immunosuppressive proteins, while increasing MHC-I expression in a panel of human melanoma cell lines. In tumor-bearing mice, combination therapy using NU7441 and the immune adjuvants stimulator of IFN genes (STING) ligand and the CD40 agonist NU-SL40 substantially increased and diversified the neoantigen landscape, antigen-presenting machinery, and, consequently, substantially increased both the number and repertoire of neoantigen-reactive, tumor-infiltrating lymphocytes (TILs). DNA-PK inhibition or KO promoted transcription and protein expression of various neoantigens in human and mouse melanomas and induced sensitivity to immune checkpoint blockade (ICB) in resistant tumors. In patients, protein kinase, DNA-activated catalytic subunit (*PRKDC*) transcript levels were inversely correlated with MHC-I expression and CD8^+^ TILs but positively correlated with increased neoantigen loads and improved responses to ICB. These studies suggest that inhibition of DNA-PK activity can restore tumor immunogenicity by increasing neoantigen expression and presentation and broadening the neoantigen-reactive T cell population.

## Abstract 2

We describe a mechanism to make cancer cells more visible to the immune system resulting in potent antitumor activity against aggressive non-responsive tumors.

## Graphical abstract

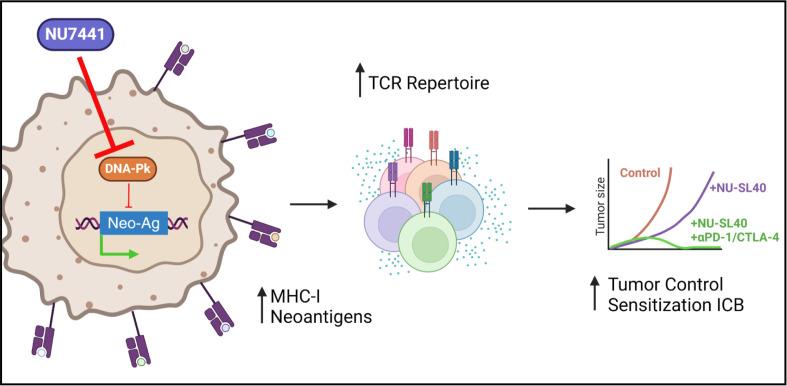

## Introduction

T cell–based cancer immunotherapies exploit the T cell’s ability to selectively recognize and destroy cancer cells while sparing noncancerous cells, representing one of the most effective cancer treatments available for different malignancies including melanoma. Recent examples include antibody blockade of checkpoint receptors (e.g., cytotoxic T lymphocyte antigen 4 [CTLA-4], programmed death 1 [PD-1], or its ligand [PD-L1]), adoptive cell transfer (ACT), tumor-infiltrating lymphocytes (TILs), or T cells engineered to express tumor-reactive T cell receptors (TCRs) (1, 2). The advances achieved through these treatments have sparked the development of newer therapies that enhance the activation of antitumor T cell responses as well as investigation into the mechanisms underlying why most patients do not benefit from single-agent immunotherapies.

Uncontrolled tumor growth in patients characterizes the immune system’s failure to recognize and/or destroy tumor cells. Often, weak tumor immunogenicity hinders the immune system’s ability to control tumor growth and arises from low surface expression of MHC-I and -II, limited expression of antigenic epitopes, or expression of antigens with low affinity for MHC. Importantly, T cells with high affinity toward self-tumor–associated antigens (TAAs) are deleted in the thymus, resulting in a repertoire of circulating T cells with limited antitumor activity. These conditions create an inadequate immune response that facilitates cancer cell growth and mechanisms of tumor immune evasion.

Immune recognition of neoantigens is a key mechanism of the potent anticancer responses observed in patients receiving immune checkpoint blockade (ICB) and ACT of TILs (3–5). Neoantigens arise from nonsynonymous somatic DNA mutations that change amino acid protein sequences. These mutated peptides are processed and loaded onto MHC-I or -II, presented on the cancer cell surface, and subsequently recognized by cytotoxic T cells (1, 6). Clinical data suggest that treatment with anti–CTLA-4 and anti–PD-1 antibodies alters and diversifies the TCR repertoire within the tumor microenvironment and is positively associated with antitumor responses (3–5). For example, studies of patients with lung cancer or melanoma undergoing ICB therapy indicate that tumors from responding patients express elevated numbers of somatic mutations (2, 7). These studies also reveal that neoantigen expression is heterogenous even within the same tumor sample; some neoantigens are clonally present in most cancer cells within the same patient, whereas other neoantigens are subclonal and expressed in a fraction of cancer cells (8). Thus, strategies targeting neoantigens are tumor specific. Although reports have highlighted that changes in the TCR repertoire can indicate antitumor activity, they have yet to elucidate how the functional capacity (cytokine production, effector function, phenotypic distinctions) associated with TCR repertoire changes correlates with antitumor immunity.

We previously screened a library of approximately 2,500 clinically relevant compounds and evaluated their ability to enhance the immunogenicity of melanoma (9) and improve DC function (10). Of these drugs, we identified several DNA–protein kinase (DNA-PK) inhibitors that enhanced MHC-I expression levels, sensitized melanoma cells to T cell–mediated killing in vitro, and enhanced the ability of DCs to activate tumor-reactive T cells including NU7441, NU7026, and KU-0060648. Of these, NU7441 was identified as the most effective. DNA-PK is a serine/threonine PK composed of a Ku heterodimer (Ku70 and Ku80) and a catalytic subunit (DNA-PKcs) that has a central role in the DNA damage response and maintenance of genomic stability (11). In this role, DNA-PK mediates ligation of DNA double-stranded breaks through nonhomologous end joining (12). At present, several therapeutic compounds are in clinical testing, assessing the antitumor efficacy of targeting DNA-PK kinase activity (NCT02516813, NCT02316197, NCT01353625, and NCT02833883). Previously, the proposed mechanisms of action were founded on the idea that DNA-PK inhibition would control tumor growth by altering DNA repair. Furthermore, several groups reported that tumor antigen expression can be upregulated by inhibiting key signaling pathways that are overly activated in melanoma (9, 13–15). Notably, we present evidence that DNA-PK inhibition has potent immunostimulatory effects on melanoma cells, as demonstrated through investigation of the mechanistic underpinnings of DNA-PK inhibition on TIL infiltration, tumor antigen expression, and TCRvβ diversity and functional capacity.

Here, we examined the combinatorial effects of treatment with NU7441 plus immune stimulation with IFN-α inducer stimulator of IFN genes (STING) agonist (STGL) and CD40 agonist in murine melanoma models on the infiltration of tumor-reactive effector CD8^+^ TILs and skewing of the tumor-reactive TCRvβ repertoire. We also revealed associations between changes in the TCRvβ repertoire and the diversification of neoantigen expression profiles in murine melanoma models and melanoma patients with melanoma. In patients with melanoma treated with anti–PD-1 and anti–CTLA-4, DNA-PK (*PRKDC)* transcript levels inversely correlated with CD8 and MHC-I transcript levels, whereas mutations in DNA-PK correlated with increased tumor mutation burden (TMB) and neoantigen load. Furthermore, while combination anti–PD-1 and anti–CTLA-4 blockade was ineffective against weakly immunogenic melanoma tumors in mice, adding DNA-PKi (NU7441) in conjunction with STGL and anti-CD40 (NU-SL40) or knocking out DNA-PK in tumors resulted in tumor regression in 75%–100% in mice. Our results suggest that DNA-PK inhibition combined with immune adjuvants enhances tumor immunogenicity by increasing neoantigen expression and presentation resulting in a broader panel of neoantigen reactive T cells with heightened functional capacity in mice and melanoma patients. This study highlights an especially relevant and promising second line therapy for individuals with tumors bearing low neoantigen loads.

## Results

### Combination DNA-PK inhibition plus immune adjuvants drive melanoma regression via a CD8^+^ T cell–dependent mechanism.

**Figure 1:**
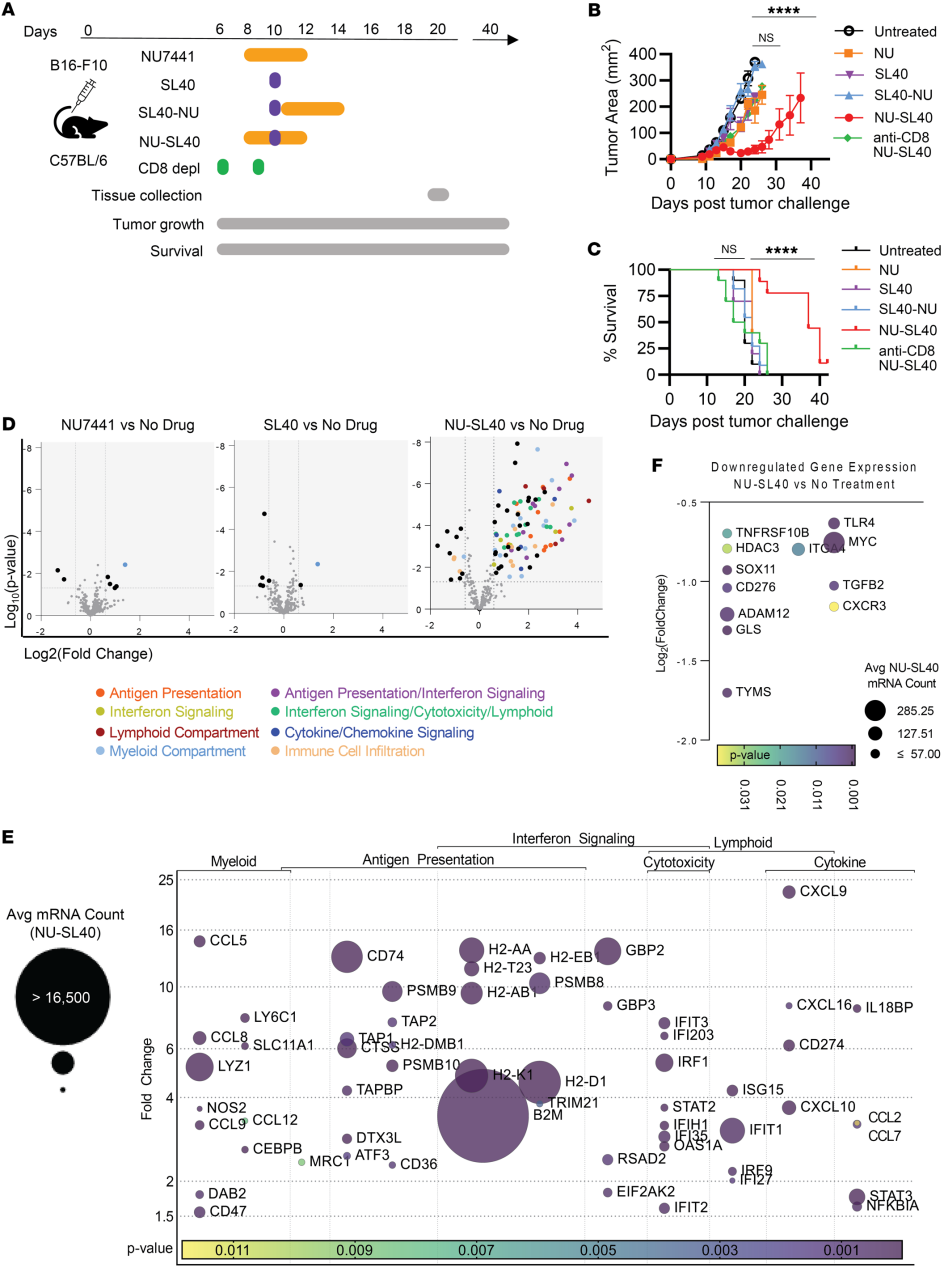
Combination immunotherapy with DNA-PK inhibition demonstrates potent antitumor CD8^+^ T cell response and is associated with a favorable antigen processing and inflammatory gene expression profile. (**A**) Schema of the treatment protocol. C57BL/6 mice with established (25 mm^2^) B16-F10 tumors underwent the treatment plans. (**A** with **B**) Tumor growth and (**C**) survival were monitored for 40 days. (**D** and **E**) Mice with established tumors were treated as described in **A**, and tumors were collected 7–9 days after initiation of treatment. (**D**) Volcano plots display the log_2_(fold change) in total mRNA transcript expression levels in B16-F10 tumors, comparing treatment versus no treatment, and the associated log_10_(*P* values) generated by NanoString gene expression analysis of 3 tumors treated with NU or SL40 or no drug and 4 tumors treated with NU-SL40. Genes in **D** are colored according to their pathway association. (**E**) Bubble plots depict the fold change in gene expression in NU-SL40–treated tumors from pathways highlighted in volcano plots as being upregulated or downregulated compared with the untreated group. Bubble size represents the average mRNA transcript counts in NU-SL40 replicates. The *P* value (compared with untreated tumors) is depicted by a color scale. ****P < 0.0001, by a mixed-effects model with Geisser-Greenhouse correction and Tukey’s multiple-comparisons test (**B**) and Kaplan-Meier survival curve with log-rank (Mantel-Cox) (**C**). Avg, average.

We previously identified the DNA-PKi NU7441 as a potent drug that decreased the expression of numerous immunomodulatory proteins, including CD55, CD73, CD155, PD-L1, and NGFR and increased HLA class I expression in vitro (9). Here, we investigated the antimelanoma activity of combination therapy using NU7441 (NU) and the immune adjuvants STING ligand plus the CD40 antibody agonist (NU-SL40) in mice bearing immunoresistant B16-F10 melanoma tumors. Female C57BL/6 mice with established tumors received treatment with either DNA-PKi (NU), STING ligand plus anti-CD40 (SL40), or the combination treatments NU-SL40 (see treatment regimen in Figure 1A). The individual treatments of DNA-PKi and SL40 alone as well as the NU-SL40 combination did not mediate substantial tumor control, resulting in tumor growth comparable to that seen in untreated mice (Figure 1B). In sharp contrast, NU-SL40 mediated tumor regression with sustained antitumor immunity and prolonged mouse survival out to 40 days (Figure 1C). As the NU-SL40 combination treatment regimen was intended to activate tumor-reactive T cells, we validated the role of CD8^+^ T cells and found that CD8^+^ T cell depletion ablated the antitumor activity of NU-SL40 therapy and reduced survival to that of untreated mice (Figure 1, B and C). Notably, B16-F10 tumors have been shown to promote cachexia characterized by weight loss, skeletal muscle wasting, and adipose tissue loss, which can be further exacerbated by immunostimulatory agents such as STING agonists and ICB (16). Despite potent antitumor immune responses, mouse weights remained similar between the treatment groups (Supplemental Figure 1A; supplemental material available online with this article; https://doi.org/10.1172/JCI180278DS1).

Altogether, these data indicate that neither DNA-PKi nor immune adjuvants alone generated productive antitumor responses. However, when combined in a specific order, NU-SL40 treatment generated effective tumor control that was dependent on the activation of tumor-reactive CD8^+^ T cell, without promoting cachexia.

### DNA-PK inhibition plus immune adjuvants induce clinically relevant gene signatures within the tumor microenvironment, including enhanced signaling in inflammatory and antigen-presenting pathways.

Several profiling studies of clinical samples from patients with cancer treated with ICB have revealed distinct gene signatures associated with response to therapy, supporting the role of these gene signatures in the diagnosis and treatment of cancer (17–19). To understand the mechanistic underpinnings that generate potent antimelanoma immune responses, we performed a PanCancer Immuno Oncology (IO) NanoString assay to profile changes to RNA in tumors from mice that received no treatment, mice treated with immune adjuvants alone, or mice that received combination treatment with DNA-PKi. Compared with untreated mice, those that received treatment with DNA-PKi or SL40 showed differentially regulated expression of 7 RNA transcripts of the 770 genes (Figure 1D, left and middle panels). In contrast, tumors from NU-SL40–treated mice showed upregulation of 87 and downregulation of 12 genes (Figure 1D, right panel). Genes differentially regulated within and between the treatment groups are shown in Supplemental Table 1.

Pathway analysis of RNAs from NU-SL40–treated mice identified gene signatures associated with IFN signaling (26 RNAs), antigen presentation (21 RNAs), lymphoid and myeloid compartments (9 and 13 RNAs, respectively), cytotoxicity (13 RNAs), and cytokine and chemokine signaling (13 RNAs), among other genes outside these pathways relevant to inflammation and anticancer pathways. Figure 1E shows a visual representation of the average transcript counts, fold changes, and *P* values in NU-SL40–treated tumors relative to untreated tumors. The greatest level of clustering was seen for genes associated with antigen presentation and IFN signaling. β2m, a key structural protein of the MHC-I molecule, had the largest RNA count in NU-SL40 tumors, indicating substantial upregulation of MHC-I. These data uphold our previous reports demonstrating that DNA-PKi increase MHC-I expression on melanoma cells and DCs (9, 10). Additionally, several H2 genes associated with antigen presentation and IFN signaling were upregulated in NU-SL40 tumors (Supplemental Table 1). Specifically, *H2-Aa* and *H2-B1* participate in processing of exogenous peptides via MHC-II and positively regulate T cell differentiation and responses to IFN-γ, respectively. *H2-K1* regulates endogenous peptide processing via MHC-I in a transporter associated with antigen processing–dependent (TAP-dependent) manner and positively regulates T cell cytotoxicity. In agreement with gene regulation favoring IFN signaling, the guanylate-binding protein (GBP) genes G*bp*2 and *Gbp3*, which are induced by IFN-γ production and have been correlated with improved overall survival in patients with cutaneous melanoma (20), were substantially upregulated in NU-SL40–treated mice. Increased expression of *Eif2ak2*, *Gbp3*, *Oas1*, *Ifit1*, *Ifit2*, *Ifit3*, and *Psmb8* — genes associated with IFN signaling and cytotoxicity — was also observed with NU-SL40 treatment, and high expression of these genes is prognostic in melanoma (21). *Cxcl9*, an antitumor-associated chemokine that facilitates recruitment of TILs to the tumor, and *Ccl5*, an inflammatory chemokine that reflects the levels of leukocyte infiltration (22), increased in expression by 22- and 16-fold, respectively, following NU-SL40 treatment. *Nos2*, a gene indicative of ROS production and typically a poor prognostic factor in melanoma (23), was the only common gene upregulated in NU-SL40, SL40, and NU treatments. NU-SL40 treatment downregulated 11 transcripts including the tumor drivers *Myc*, *Tgfb2*, *Tlr4*, *Cd276*, and *Sox11*, and genes associated with melanoma metastasis including *ITGA4*, which facilitates tumor cell migration (24–26) (Figure 1F). NU-SL40 also downregulated the thymidylate synthase *Tyms*, a critical enzyme in cell-cycle progression that is expressed at higher levels in metastatic melanoma (27).

We further evaluated changes in tumor-derived RNA associated with T cell activation. Granzyme A (*Gzma*), *Nkg7,* and CD3 subunit in NU-SL40–treated tumors were among the most upregulated genes relative to the control groups (Supplemental Figure 1B). Lower expression of GzmA in patients with melanoma treated with checkpoint inhibitors predicted an unfavorable prognosis, whereas high expression correlated with CD8^+^ T cell infiltration (28). Recently, NKG7 expression in TILs has been associated with cytotoxicity in melanoma and was found to be upregulated in tumor antigen–specific CD8^+^ TILs (29).

Collectively, these data indicate that treatment with DNA-PKi plus immune adjuvants (NU-SL40) mediated RNA profiles favoring tumor antigen processing and presentation, T cell activation, and chemokine production, all of which promote T cell recruitment.

### DNA-PKi in combination with immune adjuvants, but not alone, increases the number of activated tumor-infiltrating CD8^+^ T cells.

**Figure 2:**
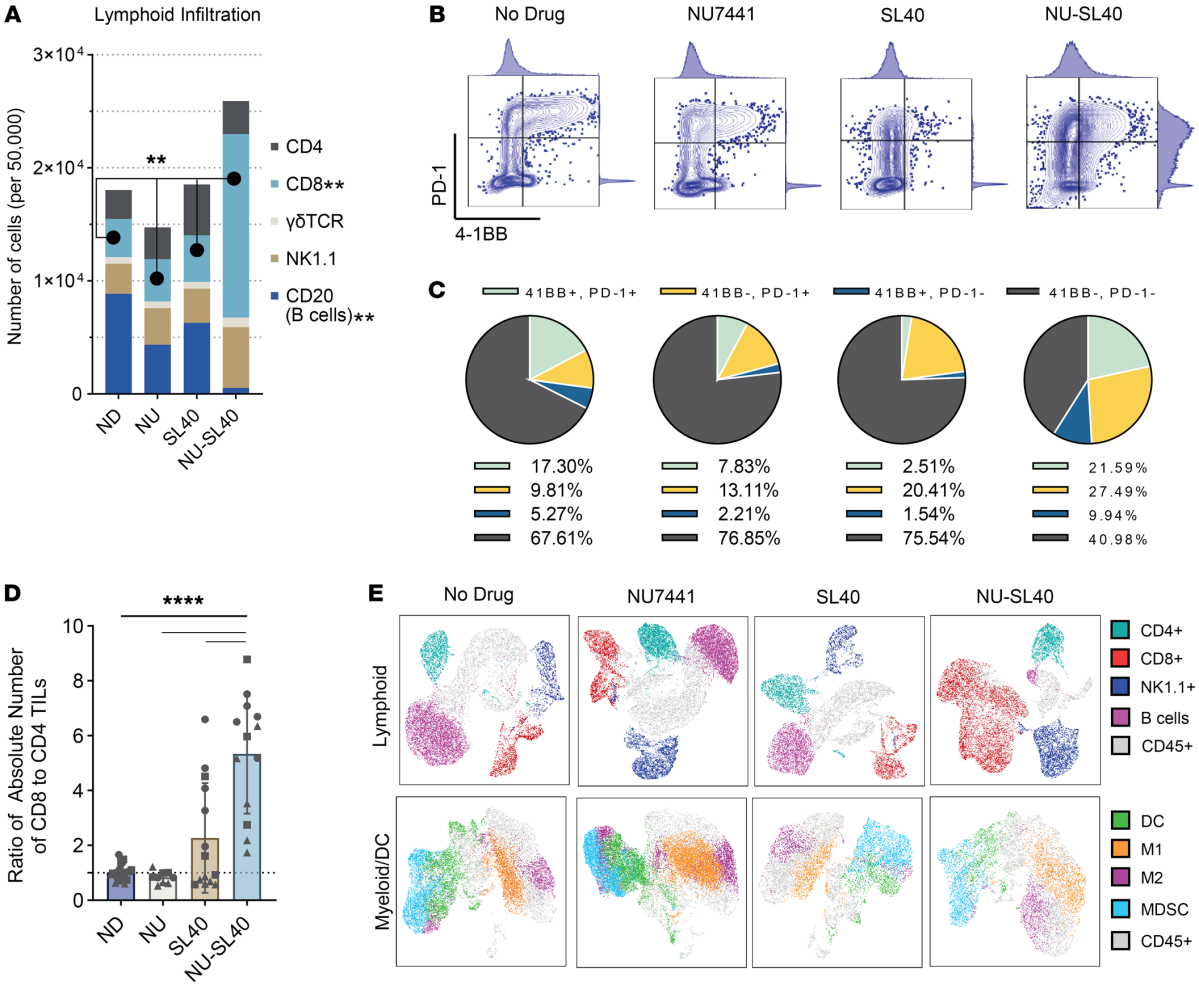
NU-SL40 treatment promotes the infiltration of activated CD8^+^ TILs and alters the tumor myeloid cell compartment. (**A**) Mice with established tumors were treated as described in Figure 1A. The indicated tumor lymphoid cell populations of single-cell, viable CD3^+^ or CD3^–^CD45^+^ cells normalized to 50,000 CD45^+^ cells were determined by flow cytometry. (no drug [ND]: *n* = 6; SL40: *n* = 5; NU: *n* = 4; NU-SL40: *n* = 5). **P < 0.01, by 2-way ANOVA. (**B**) Representative flow plots with adjunct MFI histograms and (**C**) pie charts representing the percentage of CD8^+^ TILs expressing PD-1 and/or 4-1BB across treatment groups (no drug: *n* = 5; SL40: *n* = 5; NU: *n* = 4; NU-SL40: *n* = 4). (**D**) Ratio of CD8^+^/CD4^+^ TILs (no drug: *n* = 20; SL40: *n* = 14; NU: *n* = 9; NU-SL40: *n* = 13). ****P < 0.0001, by 2-way ANOVA. (**E**) UMAP analysis of the pooled single-cell, viable CD45^+^ TIL populations (top panel) described in **A**–**C** (CD4, CD8, NK1.1, B cells) and (bottom panel) M1- or M2-like macrophages identified as CD45^+^F4/80^+^CD11c^+^CD206^–^ or CD45^+^F4/80^+^CD11c^–^CD206^–^; F4/80^+^CD45^+^CD11c^–^CD206^+^; MDSCs: CD11b^+^Gr1^+^; DC, CD45^+^CD11c^+^MHC-II^+^ (no drug: *n* = 6; SL40: *n* = 5; NU: *n* = 4; NU-SL40: *n* = 5).

To validate the RNA expression profiles suggesting an increased number of activated TILs and to further investigate changes in immune cell distribution, we quantified and phenotypically characterized tumor immune cell infiltrates (Figure 2A). We found that NU-SL40 combination treatment substantially increased the number of CD8^+^ TILs 5-fold compared with individually treated or untreated groups. NU-SL40 treatment resulted in a trend toward increased NK cell numbers, however, these changes were not statistically significant. NU-SL40 also markedly reduced the number of B cell tumor infiltrates to nearly undetectable levels (Figure 2A and Supplemental Figure 2). On the basis of the reduction of B cells in tumors in response to combination treatment with NU-SL40, we investigated how this treatment regimen altered B cell numbers in the spleen and bone marrow. We observed that, while NU-SL40 reduced B cell numbers in tumors, it increased their numbers in the spleen. In bone marrow, NU-SL40 did not affect the numbers of single-positive CD19^+^ or CD20^+^ cells but increased the number of CD19^+^CD20^+^ cells in male mice (Supplemental Figure 2). The role that B cells play in melanoma immunity is not entirely clear, as distinct subsets of B cells with contrasting functions exist, such as activating and regulatory B cells, and B cells that promote the development of tertiary lymphoid structures are present as well. However, considering the antitumor effects mediated by NU-SL40, B cells could have played a regulatory role in our study, and their reduction in number contributed to enhanced antitumor CD8^+^ T cell activity.

Surface expression of PD-1 and 4-1BB signifies cellular activation, and expression on T cells has been shown to distinguish tumor-reactive T cells (30, 31). We found that the majority of CD8^+^ TILs collected from untreated mice or from those treated with SL40 or DNA-PKi were 4-1BB^–^PD-1^–^ (Figure 2, B and C). In contrast, 59% of CD8^+^ TILs from NU-SL40–treated mice expressed either 1 or both 4-1BB and PD-1 markers and demonstrated a 3-fold increase in total PD-1^+^ and a 2-fold increase in 4-1BB^+^ single-positive cell populations when compared with untreated mice (Figure 2B and 2C, right panels). Furthermore, the expression levels of these molecules on a per-cell basis were elevated compared with levels in control groups (Figure 2B, adjunct histograms). Our data also show that NU-SL40 treatment promoted the activation and infiltration or expansion of CD8^+^ T cells to the tumor. NU-SL40 treatment induced skewing of T cell populations in favor of CD8^+^ over CD4^+^ TILs when compared with untreated mice, DNA-PKi–treated mice, and SL40–treated mice (Figure 2D).

We next performed uniform manifold approximation and projection (UMAP) analysis of CD45^+^ lymphoid and myeloid cell populations to evaluate the distribution and relationship of infiltrating immune cells in response to treatment. The lymphoid distribution in NU-SL40 treatment confirmed increases in CD8^+^ TILs, while also revealing a spatial relationship between CD8^+^ and NK1.1 cells (Figure 2, top panel). These data suggest that NU-SL40 treatment may give rise to an NK T cell population. Notably, B cells are nearly lost in NU-SL40–treated tumors. Myeloid and DC distribution (Figure 2E, bottom panel) revealed an overall decrease in myeloid-derived suppressor cells (MDSCs) in NU-SL40–treated mice.

Altogether, these data demonstrate that combination treatment, but not individual treatments, promoted the infiltration of tumor-reactive CD8^+^ T cells with a highly activated phenotype, while reducing the frequency of T cell–suppressive DCs and MDSCs.

### NU-SL40 skews CD8^+^ TIL TCRvβ diversity with increased recognition of tumor cells.

**Figure 3:**
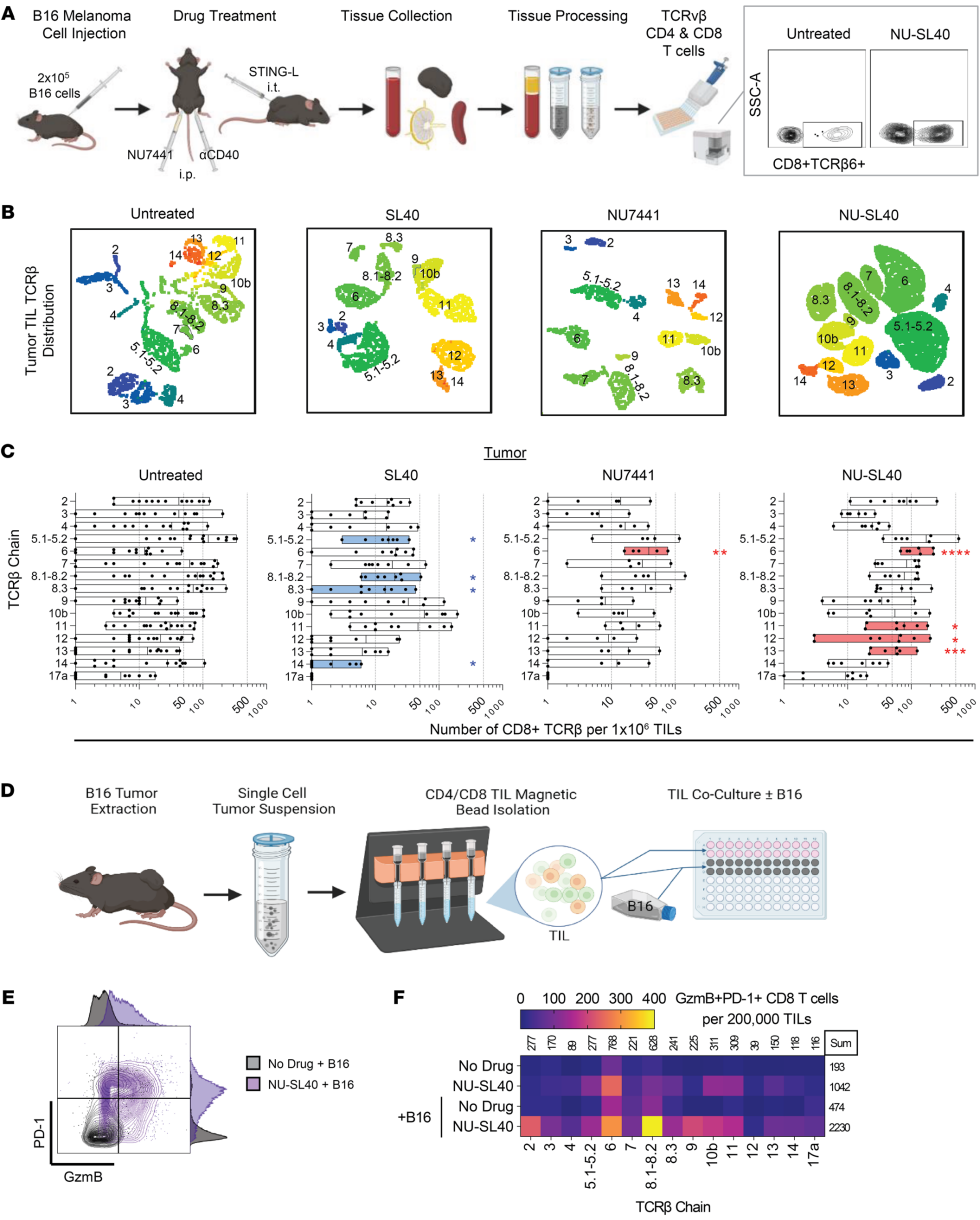
DNA-PK inhibition drives TCRvβ diversity of highly functional tumor-reactive CD8^+^ T cells. (**A**) Schematic of drug treatment and tissue processing with representative flow cytometric analysis of TCRvβ on CD8^+^ TILs. SSC-A, side scatter area. (**B**) UMAP distribution of the absolute number of CD8^+^ TILs clustered by TCRvβ chain (number labels and color scale for differentiation) (untreated: *n* = 15; SL40: *n* = 9; NU: *n* = 5; NU-SL40: *n* = 8). (**C**) Number of CD8^+^ TILs by TCRvβ chain per 1 million single-cell events. A rout outlier test was performed. Blue and red bars represent significant decreases or increases in TCRvβ counts in treatment conditions compared with no treatment. Each dot represents a single mouse (untreated: *n* = 15; SL40: *n* = 9; NU: *n* = 5; NU-SL40: *n* = 8). (**D**) Schematic of C57BL/6 B16-F10 tumor model and tumor collection for TIL isolation via magnetic bead–positive selection followed by ex vivo culturing with or without IFN-γ–pretreated (100 U/mL for 24 hours) B16-F10 melanoma cells. (**E**) Representative flow plot with adjunct MFI histograms representing the number of isolated CD8^+^ TILs expressing GzmB and PD-1 obtained from control and NU-SL40–treated mice (16-hour coculture). (**F**) Heatmap of TCRvβ distribution of CD8^+^ TILs that expressed PD-1 and produced GzmB. TILs were pooled from tumors (untreated: *n* = 4; NU-SL40: *n* = 5), and counts were normalized to 2 × 10^5^ CD3^+^ cells. The sum of the TCRvβ chain in each condition is represented above the columns, the sum of total TCRvβ in each condition is indicated to the right of each row. *P < 0.05, **P < 0.01, ***P < 0.001, and ****P < 0.0001, by multiple unpaired, 2-tailed *t* test.

Numerous studies have suggested that skewing of TCRvβ diversity in the blood and tumor following ICB is associated with better outcomes and progression-free survival (32–34). We evaluated the CD8 TCRvβ repertoire by staining 15 murine TCRvβ chains, as depicted in Figure 3A. A representative staining of CD3^+^CD8^+^TCRvβ6^+^ TILs from untreated or NU-SL40–treated mice bearing B16-F10 tumors is shown in Figure 3A (right panel). Figure 3B shows UMAP analysis of CD8^+^ TILs clustered by TCRvβ group in each treatment and demonstrates considerably larger clusters in select TCRvβ families from NU-SL40–treated tumors, indicating a substantial increase in CD8^+^ TILs relative to control groups. Figure 3C illustrates changes in the distribution of CD8^+^ TIL TCRvβ family members compared with untreated mice. In SL40–treated mice, statistically significant decreases (blue bars) in TCRvβ chains 5.1–5.2, 8.1–8.2, 9, and 14 were observed compared with the untreated group (Figure 3C). Both NU- and NU-SL40–treated mice exhibited substantial increases (red bars) in TCRvβ 6, while NU-SL40 additionally increased the frequency of TCRvβ 11, 12, and 13 compared with untreated mice (Figure 3C). We further evaluated the surface expression of TCRvβ and CD8 proteins on TILs and found that TILs from NU-SL40–treated tumors increased TCR (3- to 12-fold) and CD8 (3- to 14-fold) expression density when compared with TILs from untreated or NU-treated tumors (Supplemental Figure 3). Changes in the TCRvβ repertoire and numbers of clonally expanded circulating T cells have been shown to reflect TIL function (35). We observed that several CD8 TCRvβ family members increased in circulation following combination treatment compared with all other groups (Supplemental Figure 4). In contrast, there was a global decrease of all CD4 TCRvβ family members in the blood of SL40- and NU-SL40–treated mice (Supplemental Figure 4). However, in NU-SL40–treated mice, total CD4^+^ TIL numbers were maintained and comparable between groups (Figure 2A). In summary, NU-SL40 treatment increased the total number of CD8^+^ TILs and altered the TCRvβ repertoire of infiltrating and circulating CD8^+^ T cells. These data are clinically relevant, as a change in TCRvβ diversity is a biomarker for favorable outcomes in some cancers (36).

We next investigated the functional capacity of each CD8^+^ TIL TCRvβ family member to respond to antigenic stimulation ex vivo. CD4^+^ or CD8^+^ TILs were enriched (>98% purity) from the tumors of untreated or NU-SL40–treated mice and cultured with or without IFN-γ–stimulated B16-F10 cells (Figure 3D). We then assessed the frequency of CD8^+^ TILs producing PD-1^+^ and granzyme B (GzmB) and investigated TCRvβ usage by flow cytometry (a representative flow plot is shown in Figure 3E). In Figure 3F, the heatmap represents the sum of PD-1^+^GzmB-producing CD8^+^ TILs by expression of TCRvβ families with and without B16-F10 ex vivo stimulation. In the absence of antigen stimulation, TILs from untreated mice had relatively small numbers of PD-1^+^GzmB^+^ TILs (193 per 200,000 cells). In contrast, NU-SL40 treatment induced a 5.3-fold increase in PD-1^+^GzmB^+^ TILs (1042 cells). Among the various CD8^+^ TIL TCRvβ family members increased by NU-SL40 treatment without B16-F10 stimulation, we found the greatest increase in TCRvβ 5.1/5.2 (10-fold), TCRvβ 8.3 (14-fold), TCRvβ 10b (63-fold), and TCRvβ 11 (17-fold). In the absence of antigenic stimulation, TILs from NU-SL40–treated mice had a moderately increase (2-fold) in the number of PD-1^+^GzmB^+^ TILs and primarily belonged to TCRvβ family members 6, 10b, and 11, although most TILs from untreated mice did not produce GzmB (Figure 3, E and F). In sharp contrast, coculturing of B16-F10 tumor cells with TILs from NU-SL40–treated mice increased the numbers of PD-1^+^GzmB^+^ cells expressing TCRvβ 2 (10-fold), TCRvβ 9 (67-fold), TCRvβ 11 (8-fold), and, to a smaller degree, TCRvβ 5.1/5.2 (2-fold) and TCRvβ 8.3 (5-fold) compared with stimulated TILs from control mice (Figure 3F).

Collectively, these data highlight the ability of DNA-PK inhibition to elicit the activation of a unique group of tumor-reactive CD8^+^ T cells, increase the diversity of tumor-specific TCRvβ family members, and enhance the production of cytotoxic molecules.

### DNA-PK inhibition regulates tumor-associated antigen and neoantigen expression in mouse and human melanoma.

**Figure 4:**
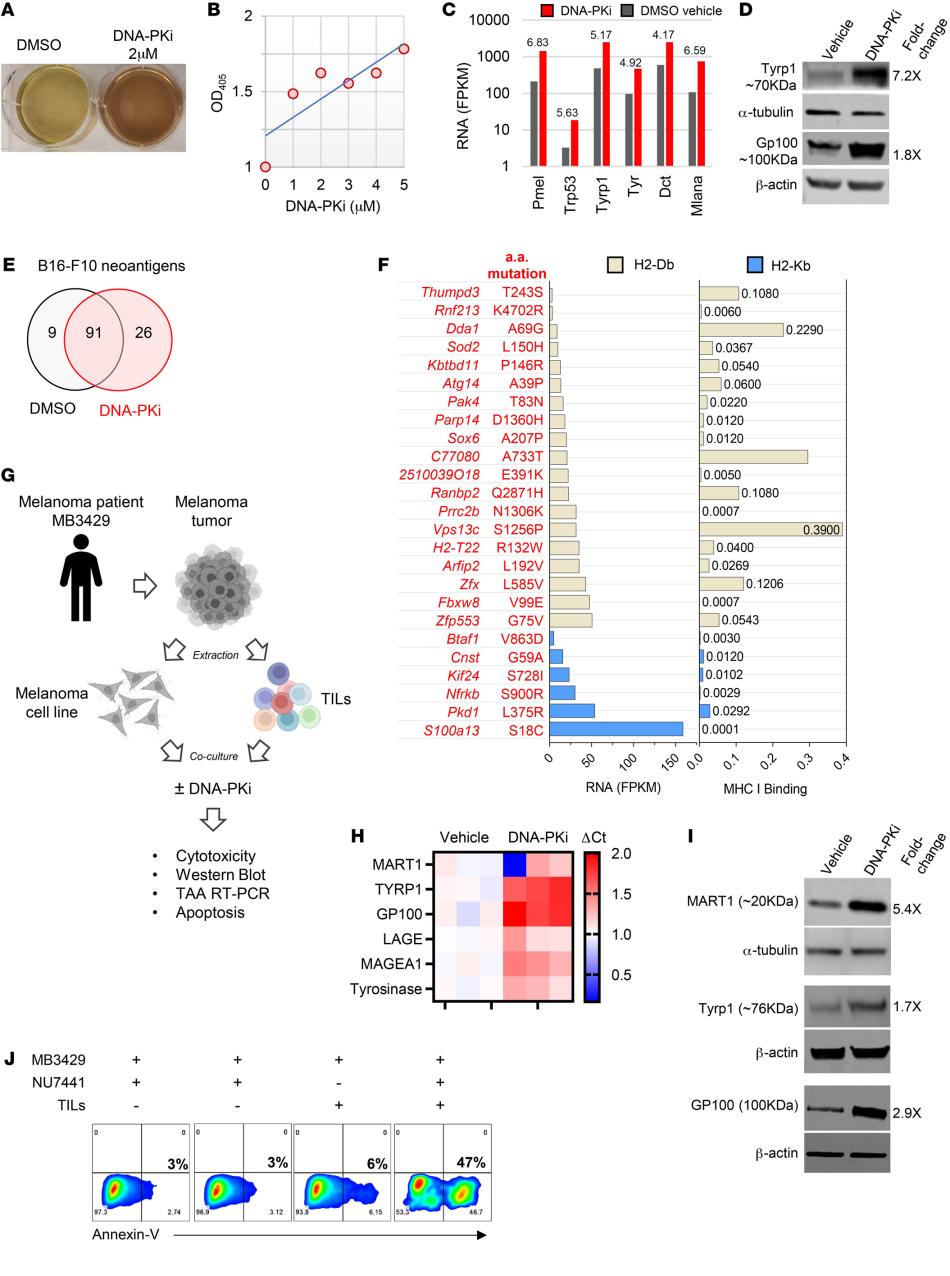
DNA-PK inhibition increases tumor-associated antigen expression levels, induces a unique neoantigen expression profile in melanoma, and represents better targets for human TILs. (**A** and **B**) B16-F10 melanoma cells were treated with 2 μM NU7441 or DMSO control for 72 hours, at which point gradual darkening was observed and the OD_405_ recorded. (**C**) Bar graph comparing the levels of RNA per FPKM of known melanogenesis-associated antigens 48 hours after treatment with 2 μM NU7441 or DMSO control. The fold change between DMSO and NU7441 treatments is noted above the bars. (**D**) B16-F10 melanoma cells were treated with 2 μM NU7441 or DMSO control for 48 hours, and the levels of the indicated proteins were determined by Western blotting. The fold change between groups is shown to the right of each band. (**E** and **F**) B16-F10 melanoma cells were treated as described in **A**, and the neoantigens and FPKM were determined as described in Methods. (**E**) Venn diagram representing the number of uniquely expressed or shared B16-F10 neoantigens present in control-treated melanomas and those induced by NU7441. (**F**) The gene name and amino acid mutation expressed following DNA-PKi treatment are shown on the left. The matched bar graph shows the levels of RNA per FPKM of neoantigen-producing genes exposed to NU7441 treatment, and the binding affinity of these epitopes for H2-Db and H2-Kb was determined using the MHC binding prediction algorithms from the Immune Epitope Database and Tools (IEDB) (iedb.org) site. (**G**) Schematic showing the generation of melanoma cell lines and TILs from a patient melanoma tumors and the experiments performed in **H**–**J**. (**H**) The MB3429 melanoma cell line was treated with 2 μM NU7441 or DMSO control for 48 hours, and the levels of the indicated transcripts were determined by RT-PCR and are shown as ΔCt. (**I**) MB3429 melanoma cells were treated with 2 μM NU7441 or DMSO control for 48 hours, and the levels of the indicated proteins were determined by Western blotting (fold change between groups is indicated to the right of each band). (**J**) Matched TILs and tumors were derived from the same tumor fragment. The tumor cell line was treated with DMSO or DNA-PKi (2 μM NU7441) for 48 hours, at which point the drug was washed off prior to coculturing with TILs at a 1:1 ratio for 18 hours. Cytotoxicity was determined by annexin V staining with flow cytometric gating on tumor cells (based on light scatter and CD3^–^) and viability dye.

While performing in vitro culturing of B16-F10 cells treated with NU7441, we observed that treatment gradually darkened cells and supernatants, suggesting an increase in melanin synthesis (Figure 4, A and B). In humans and mice, numerous proteins involved in melanin synthesis contain immunogenic CD8 epitopes that serve as TAAs (37). To better understand the transcriptional changes induced by DNA-PK inhibition in melanoma, we conducted RNA-Seq in B16-F10 melanoma cells treated with a vehicle control (DSMO) or NU7441 to explore changes to the antigen landscape, as described previously (38). We used the fragments per kilobase of exon model per million reads mapped (FPKM) to estimate gene expression in our RNA-Seq data. In agreement with predicted increases in melanin synthesis following treatment with DNA-PKi, we detected increased expression of genes in the melanin synthesis pathway that also serve as TAAs, including *Pmel* (6.8-fold), *Trp53* (5.6-fold), *Tyrp1* (5.1-fold), *Tyr* (4.9-fold), *Dct* (4.1-fold), and *Mlana* (6.9-fold) (Figure 4C). Increased RNA transcript levels were associated with increased protein expression (Figure 4D).

Upregulated expression of numerous tumor antigens following NU7441 treatment suggests that DNA-PK inhibition could regulate the transcriptional machinery in a manner that alters the expression of other genes including those coding for neoantigens. We found 91 neoantigens shared between DMSO- and NU7441-treated melanoma cells, whereas 26 unique neoantigens were induced by the DNA-PKi (Figure 4E). The mutated gene and associated changes in the amino acid sequence are shown in Figure 4F (left). The FPKM levels of NU7441-induced neoantigens and their predicted binding affinity for MHC-I (H2-Kb or Db) were also evaluated (Figure 4F, right). Consistent with the idea that DNA-PK inhibition modifies the transcriptional machinery, leading to increased transcription, we observed that NU7441 increased the transcript levels of several other shared neoantigens (Supplemental Figure 5A).

To determine whether these effects extended to human melanomas, we investigated the ability of DNA-PKi to alter the expression of clinically relevant TAAs that are currently targets for vaccine development or TCR engineering platforms. A tumor cell line with matched TILs was generated from a patient with cutaneous melanoma and cultured in the presence of DMSO or NU7441 (Figure 4G). DNA-PK inhibition increased the transcript and protein levels of numerous TAAs several-fold (Figure 4, H and I). We also investigated the direct role of DNA-PKi on TIL activity in vitro and found that at lower concentrations, DNA-PKi had no effect on IFN-γ or GzmB, but at higher concentrations, it dampened T cell activity (Supplemental Figure 5B). Despite these in vitro findings, combination DNA-PKi immunotherapy resulted in robust antitumor responses (0.125 mg/mouse/injection). We then investigated the antitumor TIL activity against a DNA-PKi–treated melanoma cell line generated from the same tumor. As shown in Figure 4J, DNA-PKi alone did not induce melanoma cell death, as measured with annexin V. Furthermore, coculturing of vehicle control–treated melanoma with paired TILs resulted in only moderate killing. In sharp contrast, pretreatment of human melanoma cells with NU7441 followed by coculturing with autologous TILs substantially increased T cell cytotoxicity.

Altogether, these data indicate that DNA-PKi alters the tumor transcriptional profile, resulting in both the induction of a unique panel of neoantigens and a simultaneous increase in the levels of various TAAs and neoantigens. The ability of DNA-PK inhibition to increase and diversify the tumor antigen landscape was associated with enhanced tumor immunogenicity, as demonstrated by improved activation and killing by tumor-reactive TILs.

### NU-SL40 treatment promotes the generation of functional neoantigen-reactive CD8^+^ TILs.

**Figure 5:**
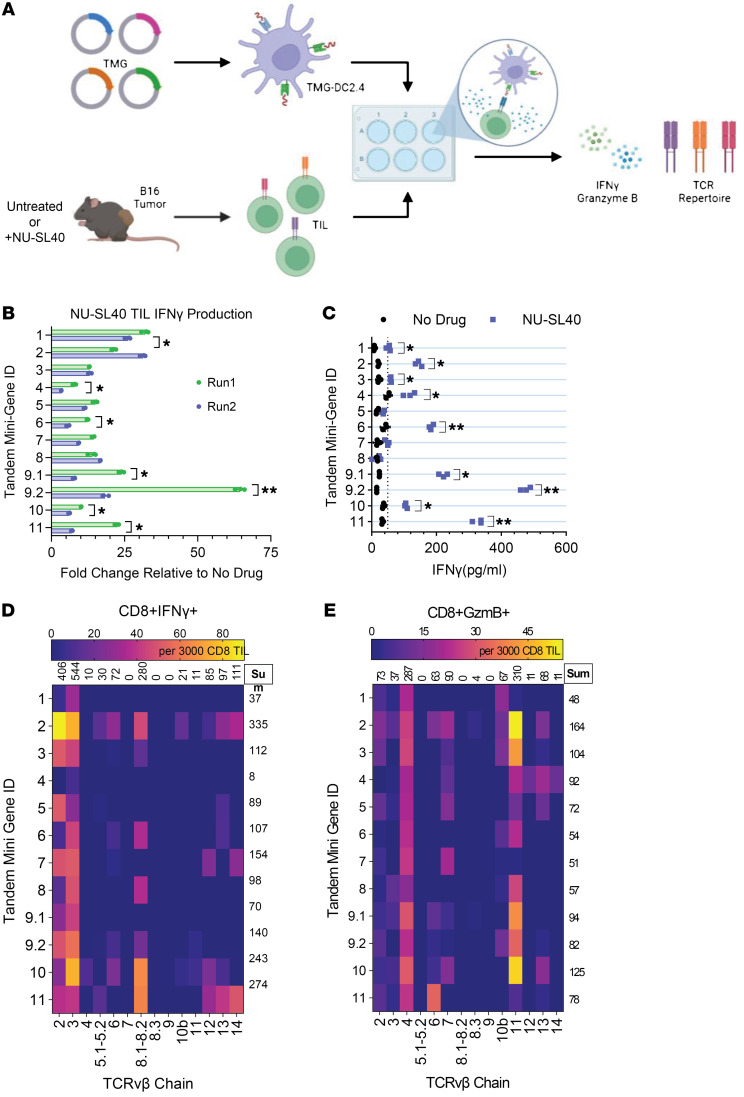
DNA-PKi plus an immune adjuvant drive the generation and expansion of a unique panel of neoantigen-reactive TILs with enhanced effector function ex vivo. (**A**) Schematic of the experimental design. Mice were treated as described in Figure 3D. TILs were isolated from NU-SL40 or untreated tumors using a positive magnetic selection for CD4^+^ and CD8^+^ T cells. Twelve plasmids were generated to contain TMGs of the 10 neoantigens identified in Figure 4. (**B** and **C**) TMGs were transfected into the murine DC2.4 line and cocultured with CD4^+^ and CD8^+^ TILs collected from control- or NU-SL40–treated mice (pooled from 10 mice/group) at a 1:10 TIL/DC ratio. After 48 hours, IFN-γ production by TCRvβ-specific responses to DC-presented neoantigens was determined by ELISA. Bar graphs depict IFN-γ production by TILs stimulated with TMG-DCs compared from 2 independent experiments. Values were normalized to production after stimulation with a TMG-GFP control. (**D** and **E**) The ability for CD8^+^ TILs to produce IFN-γ or GzmB was determined by intracellular staining and flow cytometry. TCRvβ usage in response to stimulation with each TMG-expressing DC was also investigated. Heatmaps represent the number of CD8^+^ TIL per 3,000 total TIL expressing different TCRvβ chains and producing (**D**) IFN-γ or (**E**) GzmB in response to stimulation from each TMG.

Considering that TILs from NU-SL40–treated tumors exhibited an increased response following B16-F10 stimulation (Figure 3) and that DNA-PKi increased neoantigen expression (Figure 4), we characterized the ability for TILs to recognize a panel of the NU7441-induced neoantigens described in Figure 4. CD3^+^ TILs isolated from untreated and NU-SL40–treated tumors were activated using mouse DCs engineered to express tandem minigenes (TMGs) coding for various neoantigens, as described previously (39) (Figure 5A). Each TMG codes for 10 neoantigens, and each neoantigen contains 15 amino acids downstream and upstream of the mutations (39, 40). We observed that TILs isolated from NU-SL40–treated mice were sensitive to several TMG-expressing DCs and produced substantially higher quantities of IFN-γ as compared with TILs from untreated tumors (Figure 5B). Notably, we detected the induction of IFN-γ in TMG-DC–stimulated NU-SL40 TILs compared with untreated TILs between 2 different experiments, and although the intensity of responses varied, the overall trend remained consistent (Figure 5C). These changes in CD8^+^ TIL effector responses to different neoantigens implies an evolving tumor antigen landscape in which NU-SL40 is capable of differentially activating TILs with distinct TCRvβ expression profiles.

We next performed flow cytometry to investigate the ability of specific CD8^+^ TIL TCRvβ family members to become activated by neoantigens, as assessed by IFN-γ and GzmB production. The heatmaps in Figure 5, D and E, summarize the number of TILs and specify the TCRvβ family members that produced IFN-γ and GzmB in response to stimulation with different TMGs. The numbers above each column are the sum of cytokine-producing TILs per TCRvβ family member, whereas the sum of cytokine-producing TILs responding to specific TMGs is indicated by row. We observed that TILs expressing TCRvβ 2, 3, and 8.1/8.2 demonstrated the greatest degree of response against a broad array of TMGs based on IFN-γ production relative to a GFP-TMG control (Figure 5D). In contrast, we did not detect appreciable numbers of IFN-γ–producing TCRvβ 4, 8.3, 9, 10b, or 11 TILs. Individually, each TMG prompted cytokine production by a limited number of TCRvβ family members (Figure 5D). For example, TMG4 did not induce cytokine production, whereas TMG1 only provoked TCRvβ 3 TILs to produce IFN-γ. However, TMG2, TMG9.1, TMG9.2, TMG10, and TMG11 elicited robust IFN-γ production from numerous TCRvβ groups, while TMG3, TMG7, and TMG9.2 activated TILs to a lesser extent.

We also evaluated the ability of TMG-DCs to elicit GzmB production by TILs from NU-SL40–treated mice. The most responsive TCRvβ family members were TCRvβ 4 and TCRvβ 11, which accounted for 64% of responding TILs, followed by TCRvβ 6 and TCRvβ 2. TMGs 2, 3, and 10 stimulated 29% of GzmB-producing TILs (Figure 5E). Most TMGs promoted GzmB production by at least 2 TCRvβ family members, with TMGs 2, 3 and 10 stimulating 38% of T cells. Notably, the TCRvβ family members that produced GzmB differed from those that produced IFN-γ. Specifically, the greatest number of GzmB–producing TILs belonged to the TCRvβ families 4 and 11, whereas TCRvβ 2, 3, and 8.1/8.2 predominately produced IFN-γ.

Together, these findings highlight DNA-PKi’s ability to increase the number of functionally active TIL populations and promote a more versatile TCRvβ repertoire reactive against a broader, diverse panel of neoantigens. These data also underscore the generation of a distinct subset of neoantigen-reactive TILs capable of exclusively producing IFN-γ or GzmB against different neoantigens.

### Alterations in DNA-PK gene expression and sequence in patients with melanoma treated with checkpoint immunotherapy correlate with CD8^+^ TIL infiltration, neoantigens loads, and responses to therapy.

**Figure 6:**
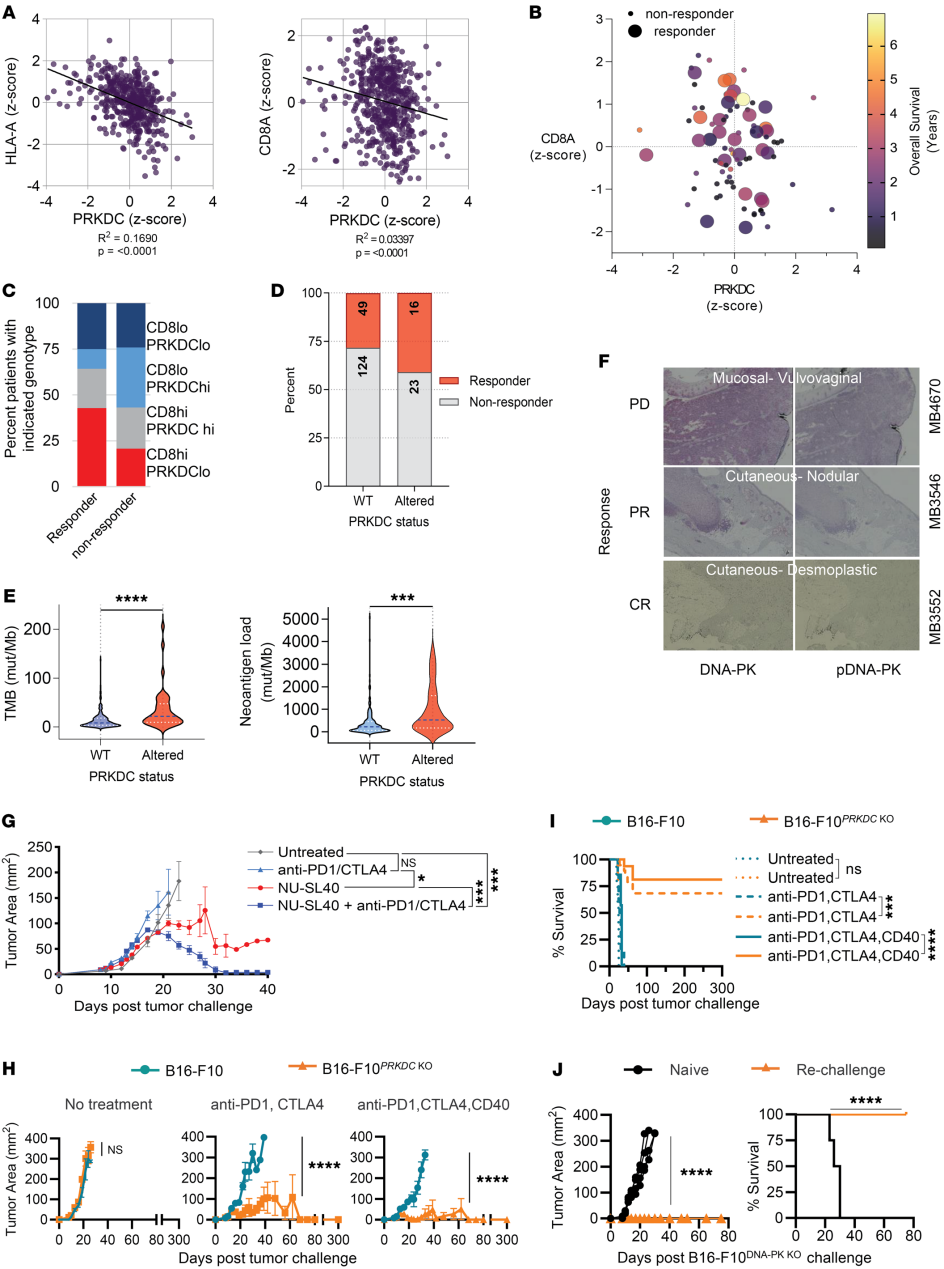
*PRKDC* levels inversely correlated with TILs, MHC-I, and the response to ICB therapy in patients with melanoma and are mirrored by B16-F10*^PRKDC^* ^KO^ tumors. (**A**) Scatter plot of *z* scores for HLA-A and CD8α expression versus *PRKDC* expression obtained from TCGA. (**B**) Associations between CD8α and *PRKDC* mRNA expression by *z* score, with overall survival in months indicated by the color scale in patients who were responders (large circles) or nonresponders (small circles). (**C**) Graph distinguishing the percentage of CD8^lo^, CD8^hi^, PRKDC^lo^, and PRKDC^hi^ cells in melanoma tumors that responded or not to ICB. (**D**) Percentage of patients with melanoma expressing WT or altered *PRKDC*, who responded or not to ICB. (**E**) Violin plots depicting differences in tumor mutation burden (left, *P* < 0.0001) and neoantigen load (right, *P* = 0.0002) in patients with normal (WT, *n* = 172) versus altered (*n* = 40) *PRKDC* expression. Statistical significance was determined by unpaired Mann-Whitney *U* test. (**F**) Staining for total DNA-PK and p–DNA-PK (Ser2056) in samples from patients with melanoma. (**G**) C57BL/6 mice with established (25 mm^2^) B16-F10 tumors remained untreated or were treated with anti–PD-1/–CTLA-4 blockade, NU-SL40, or NU-SL40 in conjunction with anti–PD-1/–CTLA-4 blockade (*n* = 8/group). Tumor growth was monitored over time. (**H** and **I**) WT B16-F10 cells (orange) and melanoma cells engineered to KO *PRKDC* (teal) were injected into mice. When tumors were established, mice were left untreated or treated with anti–PD-1/–CTLA-4 with or without anti-CD40 therapy. (**H**) Tumor growth and (**I**) survival were monitored over time (*n* = 8 mice/group). (**J**) Mice treated with combination anti–PD1/–CTLA-4 and anti-CD40 that showed controlled tumor growth were rechallenged with DNA-PK–KO cells after 300 days (naive, *n* = 4; rechallenge; *n* = 5). Tumor growth and survival were monitored among rechallenged and naive, challenged mice using 2-way ANOVA. *P < 0.05, ***P < 0.001, and ****P < 0.0001.

In melanoma, CD8^+^ TIL infiltration has been positively associated with MHC-I expression levels, a high TMB, and neoantigen loads (41), as well as a response to checkpoint inhibitors. A recent report by Tan et al. demonstrated that mutations in *PRKDC* could serve as predictive biomarkers for positive outcomes with ICB in gastric cancers (42). Thus, we reviewed publicly available exome-sequencing data from patients with melanoma undergoing CTLA-4 or PD-1 blockade therapy to investigate potential correlations between *PRKDC* levels and response to checkpoint therapy (2, 33, 34). To uncover associations between CD8 infiltrates and the expression of MHC-I (*HLAA*) with *PRKDC* (DNA-PK) levels, we analyzed melanoma patient data from The Cancer Genome Atlas (TCGA). Both increased *CD8a* and *HLA-A* expression negatively correlated with *PRKDC* expression, suggesting that decreased DNA-PK expression and activity may promote CD8 tumor infiltration (Figure 6A). We observed that patients who responded to immunotherapy tended to have higher *CD8a* expression, with a trend toward longer overall survival seen with lower *PRKDC* expression (Figure 6, B and C).

The *PRKDC* gene encoding DNA-PKcs is a critical component of the DNA damage repair (DDR) pathway, and mutations in the tumor DDR pathway can serve as important biomarkers for a response to checkpoint-based immunotherapies. To further understand how alterations in the *PRKDC* gene correlated with response to immune checkpoint inhibition, we analyzed data from three melanoma clinical trials utilizing PD-1/CTLA-4 therapy (2, 33, 34) and found that a higher percentage of patients with altered (mutations, deletions, amplifications) *PRKDC* demonstrated superior responses to immune checkpoint inhibition (Figure 6D). We further analyzed the exome sequencing data set for Tumor Mutation Burden (TMB) and Neoantigen Load and categorized patients for *PRKDC* expression as either normal (WT) or altered. We found that patients with *PRKDC* alterations had higher TMB and neoantigen load (Figure 6E). The increased TMB and neoantigen load in melanoma patients with *PRKDC* mutations or deletions supports our findings that DNA-PKi not only increases the expression of neoantigen transcripts but also induces what we considered to be a new panel of neoantigens.

As phosphorylation regulates the activity DNA-PKcs, we utilized immunohistochemistry to investigate the total and phosphorylated levels of DNA-PKcs (p–DNA-PKcs) in patients with melanoma undergoing ICB therapy and their response to treatment. The data in Figure 6F show a mucosal-vulvovaginal melanoma sample with elevated levels of total and p–DNA-PK (Ser2056) from a patient that experienced progressive disease following combination checkpoint therapy. In contrast, melanoma expressing moderate levels of DNA-PK, but deficient or low levels of p–DNA-PK demonstrated favorable responses to checkpoint therapy.

### DNA-PKi confers PD-1/CTLA-4 ICB efficacy against established B16-F10 melanoma tumors.

B16-F10 melanoma is an extremely aggressive cell line, in part owing to its weak immunogenicity. Combination blockade of CTLA-4 and PD-1 on their own are insufficient for controlling tumor growth (43). As shown in Figure 1, a single round of NU-SL40 therapy, in the absence of ICB, achieved tumor regression in 100% of mice but this response was transient, and all mice succumbed to the tumor within approximate;y 40 days. The standard of care for patients with melanoma is combination blockade of CTLA-4 and PD-1 and results in a 5-year overall survival of approximately 60%. Since the efficacy of checkpoint therapy is linked to the neoantigen load and NU7441 increased neoantigen expression, we investigated the ability for NU-SL40 to enhance the antitumor activity of combination treatment with anti–PD-1/–CTLA-4 in mice bearing an established B16-F10 tumor. Administration of NU-SL40 delayed tumor growth whereas mice treated with ICB sustained similar growth kinetics to untreated mice (Figure 6G). In sharp contrast, mice treated with NU-SL40 and anti–PD-1/–CTLA-4 exhibited tumor regression in all mice. Despite the association of B16-F10 tumors to promote cachexia in the setting of immunostimulation and robust antitumor immune responses, there was no marked variation in mouse weights between treatment groups in our model (Supplemental Figure 6).

To determine the role that DNA-PK in cancer cells played in altering their immunogenicity, we knocked out DNA-PK in B16-F10 melanoma cells (B16-F10^DNA-PK–KO^) (Supplemental Figure 7, A and B) and investigated mouse survival and tumor growth in response to checkpoint therapy. In the absence of treatment, DNA-PK deletion had no effect on tumor growth (Figure 6H, left). However, B16-F10^DNA-PK–KO^ tumors were sensitized to anti–PD-1/–CTLA-4 therapy (Figure 6H), middle panel. Our data in Figure 1, indicated that including anti-CD40 treatment to activate APCs contributed to generating antitumor T cell responses. Thus, we sought to determine whether adding anti-CD40 treatment further improved tumor immunity against B16-F10^DNA-PK–KO^ tumors. As shown in Figure 6H (right panel), supplementing with anti-CD40 substantially enhanced antitumor responses against B16-F10^DNA-PK–KO^ tumors but not control tumors. Immunological responses were robust, and mice remained tumor free for 300 days (Figure 6, H and I). To examine whether this combination treatment induced long-lived T cells capable of controlling a subsequent tumor rechallenge, surviving mice and a group of naive mice were injected with B16-F10^DNA-PK–KO^ cells, and tumor growth kinetics and survival were monitored for 75 days. All surviving mice demonstrated a vigorous antitumor response capable of controlling tumor growth (Figure 6J). In sharp contrast, all naive mice succumbed to tumor challenge.

Collectively, these data show that reduced *PRKDC* levels were associated with increased *HLAA* expression, TIL CD8 expression, and an improved response to checkpoint therapy. Furthermore, inhibiting DNA-PK with a pharmacological inhibitor or knocking it out induced efficacy of ICB in a typically immunotherapy-resistant melanoma tumor.

## Discussion

Despite serving as the first-line treatment for melanoma, combination therapy with PD-1 and CTLA-4 blockade was ineffective in 40% of treatment-naive patients with melanoma. The lack of a durable response or recurrence of tumors demonstrated selective pressures impairing the immune system’s recognition and destruction of the tumor, leading to the outgrowth of cancer cells with reduced MHC-I expression or limited expression of antigenic epitopes. Collectively, our studies highlight that (a) DNA-PKi enhanced antitumor immune responses by creating a potent inflammatory tumor environment that favored tumor antigen presentation; (b) DNA-PKi increased the levels and induced the expression of additional neoepitopes that activated a broad panel of neoantigen-reactive T cells with potent tumor-killing activity; (c) reduced *PRKDC* (DNA-PK) levels inversely correlated with CD8 tumor infiltration and elevated MHC-I expression; (d) *PRKDC* mutations were associated with higher TMB and neoantigen loads in human melanomas and enhanced responses to ICB; and (e) KO of DNA-PK in mouse melanoma tumors conferred sensitivity to ICB and sustained tumor regression.

Approaches designed to diversify and increase the neoantigen landscape or to intensify the expression of TAAs are especially relevant to cancers with a low TMB, including rare melanoma subtypes such as uveal, mucosal, and acral melanomas. Neoantigen-reactive T cells play a critical role in destroying tumors in patients receiving ICB and ACT (3–5). Studies of patients with lung cancer and melanoma undergoing ICB therapy highlighted a correlation between the response to therapy and the number of tumor somatic mutations (2, 7). These studies also revealed that neoantigen expression is heterogenous: while some neoantigens are clonal and present in most or all cancer cells within the same patient, other neoantigens are subclonal and expressed in only a fraction of the cancer cells (8). In our studies, DNA-PKi alone was sufficient to drive the expression of various TAAs and neoantigens. We observed a significant delay in tumor growth in all mice and complete tumor regression in approximately 37% of mice treated with DNA-PKi plus immune adjuvants, and 100% tumor regression when paired with anti–PD-1/–CTLA-4 blockade. In contrast, none of the mice treated with any other combination of DNA-PKi or ICB exhibited tumor regression. These data suggest that diversifying and increasing the expression of neoantigens contributed to the induction of effective T cell–mediated tumor immunity. Thus, development of therapies targeting neoantigens can generate tumor-specific immune responses. Moreover, boosting immune responses toward the generation of known TAAs is also advantageous, as they can be highly expressed across patients. In isolates from TILs, peripheral blood, or lymph nodes (44), the frequency of TAA-specific T cells is higher than that of neoantigen-reactive T cells. Furthermore, whereas neoepitopes and the presence of neoepitope-specific T cells vary among patients with a common cancer type, the same TAAs such as MART-1, GP100, TYRP, etc., are routinely expressed in diverse types of cancer (45). Our studies suggest that DNA-PK inhibition is a potential strategy to boost TAA and neoantigen-reactive T cell responses and improve the efficacy of anti–PD-1/–CTLA-4 blockade–based therapies.

The mechanisms by which DNA-PK inhibition drives the expression of neoantigens and TAAs is not clear. DNA-PK is a serine/threonine PK with a vital role in the DNA damage response and maintenance of genomic stability by mediating ligation of DNA double-stranded breaks through nonhomologous end joining (11, 12). However, relevant to our studies, and independent of its role as a DNA repair enzyme, emerging evidence suggests that the DNA-PKcs can play a critical role in transcriptional regulation. For example, Goodwin et al. demonstrated that in prostate cancer, DNA-PK interacted with the androgen receptor (AR) at DNA transcriptional sites where it facilitated AR-dependent transcriptional transactivation (46) of a panel of genes that drive prostate cancer progression. In melanoma, Kotula et al. demonstrated that DNA-PKcs enhances prometastatic activity by promoting the transcription of genes coding for secreted proteins known to modulate tumor migration and invasion (47). Giffin et al. demonstrated that DNA-PK, via the Ku subunits, binds directly to NRE1 DNA sequence elements within the mouse mammary tumor virus (MMTV) promoter, resulting in transcriptional repression (48). DNA-PK has also been demonstrated to bind to the E-box/TATA DNA elements and suppress gene expression (49).

In support of DNA-PK’s role as a transcriptional repressor in our model, we found that inhibiting DNA-PK drove neoantigen and TAA expression at the transcriptional level. Furthermore, in patients treated with anti–PD-1/–CTLA-4 blockade therapies, reduced *PRKDC* levels inversely correlated with CD8^+^ TIL and MHC-I expression, and *PRKDC* mutations were associated with higher neoantigen loads and enhanced responses. On the basis of these reports and in conjunction with our data demonstrating the increased transcription of a variety of genes with neoantigens, our ongoing studies are focused on understanding whether DNA-PK plays a role as a transcriptional repressor and whether blocking this function contributes to the restoration of tumor antigen expression.

The identification of baseline biomarkers to predict clinical outcomes or safety has become a priority for administering cancer immunotherapies. Among these biomarkers are CD8^+^ TILs displaying specific inflammatory cytokine profiles. Our data indicate that genes coding for the TIL-recruiting chemokines CXCL9 and CCL5 were among the most upregulated genes in NU-SL40–treated mice. Other biomarkers included microsatellite instability status and TMB that could serve as a surrogate for the presence of T cell epitopes derived from neoantigens. Our data reveal that in patients with melanoma who received anti–PD-1/–CTLA-4 blockade, *PRKDC* mutations were associated with a higher TMB, neoantigen loads, and enhanced responses. Other potential biomarkers associated with the presence of tumor-reactive T cells and response to immunotherapies include proteins that regulate antigen processing and MHC expression (50). Our data indicate that in patients with melanoma, reduced *PRKDC* levels correlated with increased CD8^+^ TILs and MHC-I expression, both of which are strong indicators of a response to immunotherapy. Furthermore, H2 family members that participate in antigen processing and presentation by MHC-I and -II were upregulated in response to NU-SL40 treatment. DNA-PKi was previously reported to reduce the expression of PD-L1 and several other immunomodulatory proteins, while increasing MHC-I expression in a heterogeneous panel of melanoma cell lines (9). Finally, IFN-γ gene signatures, including the presence of IFN-γ in the circulation, in the tumor, or relating to the responsiveness of tumors to IFN-γ, have also been suggested to be relevant biomarkers. Our studies demonstrate that administration of a DNA-PKi in conjunction with immune adjuvants strongly induced a clinically relevant IFN-γ and inflammatory gene signature favoring tumor antigen processing and presentation and T cell recruitment. We propose that checking for DNA-PK transcript or protein levels or the presence of mutations in *PRKDC*, alone or in combination with existing biomarkers, could improve the reliability of predictive indicators of a response to T cell–based cancer immunotherapies.

Through these studies, we propose that DNA-PK inhibition plays an alternate role as an immune-modifying agent through its ability to promote an inflammatory tumor environment and positively affect the neoantigen load and TAA expression. In concert with its ability to promote antigen processing, DNA-PK inhibition can broaden the repertoire of neoantigen-reactive T cells with heightened antitumor activity. Numerous compounds are in clinical testing to evaluate the efficacy of targeting DNA-PK (NCT02516813, NCT02316197, NCT01353625, and NCT02833883) and could offer the opportunity to design clinical trials around the concept of inhibiting DNA-PK activity to promote tumor immunogenicity in the setting of therapy-resistant tumors.

## Methods

### Sex as a biological variable.

Our study analyzed data from male and female patients and used male and female mice.

### Cell culture.

B16-F10 cells (CRL-6475, ATCC) were cultured as recommended by American Type Culture Collection (ATCC). For in vitro experiments, B16-F10 cells with 70% confluence were stimulated with 100 U/mL recombinant mouse IFN-γ (575304, BioLegend) 16–20 hours before collection. DC2.4 murine DCs (32011203, MilliporeSigma) were cultured in RPMI 1640 supplemented with 1× l-glutamine (TMS-002-C, MilliporeSigma), 1× NEAA (TMS-001-C, MilliporeSigma), 1× HEPES (15630080, Gibco, Thermo Fisher Scientific), and 0.0054X β-mercaptoethanol (ES-007-E, MilliporeSigma). Isolated TILs and PBMCs were cultured in TIL media (RPMI 1640 supplemented with 10% FBS, 1% penicillin-streptomycin [15070063, Thermo Fisher Scientific], 50 U/mL IL-2 [NDC 65483-116-07, Proleukin, Aldesleukin]), and 50 mM β-mercaptoethanol). Cell lines were evaluated weekly for mycoplasma. Adherent cell lines were harvested with 0.25% Trypsin/EDTA (25200056, Thermo Fisher Scientific). All cells were cultured at 37°C, 7% CO_2_.

### Animal model.

Eight- to 10-week-old C57BL/6J mice (The Jackson Laboratory, JAX no. 000664) were used for all experiments involving B16-F10 melanoma cell injections. Mice were humanely euthanized using compressed CO_2_ air for primary euthanasia and cardiac perfusion or cervical dislocation for secondary methods.

### Therapeutics.

NU7441 (S2638-05, SelleckChem; HY-11006 5 mg, MedChemExpress) was reconstituted in warm DMSO at 12.5 mg/mL, creating 10× aliquots, and then diluted to 1× at 1.25 mg/mL with 5% kolliphor (C5135-500G, MilliporeSigma) in saline. STING ligand (DMXAA – tlrl-dmx, InvivoGen) was reconstituted in DMSO at 10 mg/mL, creating 2× STGL aliquots, and then diluted to 1× in molecular-grade H_2_O to 5 mg/mL. Anti-CD40 antibody (BE0016-2, Bio X Cell) was suspended at 1 mg/mL in sterile saline. Anti-CD8 depletion antibody (clone 53-6.7; Bio X Cell) was suspended at 1 mg/mL in PBS.

### In vivo tumor model and drug treatment.

Eight- to 10-week-old mice were injected s.c. in the flank, lateral to the midline, with 2 × 10^5^/100 μL B16-F10 melanoma cells in 0.1% FBS in PBS. For DNA-PKi alone and NU-SL40 combination, NU7441 was administered i.p. twice a day (9 hours between treatments) for 5 days in 100 μL (0.125 mg/mouse/injection) when tumors were approximately 25 mm^2^ in size. When tumors reached approximately 40 mm^2^ in size, STING ligand was administered once intratumorally (i.t.) in 10 μL (50 μg/mouse). In vivo mouse anti-CD40 antibody was administered i.p. once in conjunction with STING ligand injection in 100 μL (100 μg/mouse). For NU-SL40, SL40 was administered when the tumors reached approximately 40 mm^2^ in size, followed by 5 days of once-daily NU7441 administration. For CD8 depletion, mice were injected i.p with 100 μL (100 μg/mouse) anti-CD8 antibody at 4 and 2 days prior to SL40 injections. Mice were euthanized and tumors were harvested 7–9 days from the initiation of NU7441 treatment.

### Tumor and lymph nodes.

B16-F10 tumors and draining inguinal lymph nodes were extracted and mechanically digested through a 70 μM strainer into HBSS wash buffer (5 mM EDTA, 2% FBS). The tumor single-cell suspension was then resuspended at 10 mL/gram tissue in digestion buffer consisting of RPMI 1640 and 2U TURBO DNase (AM2238, Invitrogen, Thermo Fisher Scientific) and 100 μL Liberase DH (5401054001, MilliporeSigma). The tumor suspension was incubated and rocked at 37°C for 30 minutes and then poured through a 40 μM strainer.

### Blood collection and spleen extraction.

Blood was collected via the cardiac perfusion secondary euthanasia method, and spleens were extracted. Blood was collected in LH lithium heparin tubes (450477, Greiner Bio-One) and held on ice. Spleens were mechanically processed through a 70 μM strainer into wash buffer. Blood and splenic pellets were resuspended in 1× RBC Lysis Buffer (420301, BioLegend) for 2 minutes and quenched with PBS, and then prepared for antibody staining or resuspended in 1 mL TIL media and plated in a 48-well plate for incubation overnight.

### Serum.

Blood was collected into microcentrifuge tubes, held on ice while coagulating for 75 minutes, and then centrifuged at 1,000*g* for 10 minutes to isolate serum. Serum was stored at –80°C.

### RNA extraction from tumor tissue.

Tumor tissue (<1 g) was placed into RNase free microcentrifuge tubes without buffer, on ice. Tumor cells were lysed, and RNA and DNA were extracted from tissue using an RNeasy Mini Kit (74104, QIAGEN). Sample concentration and purity were determined using NanoDrop One/OneC (Thermo Fisher Scientific), and RNA integrity was further validated using RNA ScreenTape for the Agilent 2200 TapeStation (5067-5576, Agilent Technologies).

### Ex vivo–isolated TIL B16-F10 rechallenge.

TILs from a digested single-cell tumor suspension were isolated using mouse CD4, CD8 (TIL) MicroBeads (130-116-480, Miltenyi Biotec) and LS columns (130-042-401, Miltenyi Biotec), per the manufacturers protocol. TILs were suspended in TIL media and cultured in a 96-well plate alone or with stimulated B16-F10 cells (1:1 ratio, 200,000 total/200 μL per well) for 20 hours for surface/intracellular staining. Supernatant was collected at 14 hours followed by GolgiStop (BD) incubation for 6 hours. Detailed procedures are available in the Supplemental Methods.

### NanoString gene expression.

Samples were prepared for RNA hybridization by diluting RNA to 15 ng/μL in RNase-free water. The NanoString Gene Expression CodeSet RNA Hybridization protocol was followed to hybridize RNA to the nCounter Mouse PanCancer IO 360 Panel Codeset (XT-CS0-MIO360-12, NanoString) and run on nCounter Sprint. Samples were analyzed using nSolver and ROSALIND (https://rosalind.bio/) analysis platforms with normalized fold changes and *P* values as described in the nCounter Advanced Analysis 2.0 User Manual.

### Melanoma tumor antigen expression.

RNA transcript and protein expression of selected tumor-associated antigens was determined in mouse and human melanoma cells by real-time PCR (RT-PCR) or Western blotting. Cells (5 × 10^5^) were treated with 4 μM NU7441 in 6-well plates and harvested 48–72 hours later.

### IHC.

Tissues were stained according to established protocols at the University of Colorado Histology Core using DNA-PK (12311, Cell Signaling Technology) rabbit mAb at 1:100 and p–DNA-PK (ab18192, Abcam) rabbit polyclonal at 1:200.

### Neoantigen identification and TMG neoantigen plasmid generation.

Neoantigens were determined as previously described (37). B16-F10 cells were treated in tissue culture with 2.5 μM NU7441 for 48 hours, at which time RNA and genomic DNA were extracted. As controls, RNA and genomic DNA were extracted from the spleens of C57BL/6 mice. Whole-exome sequencing (WES) data were analyzed by the standard Exome Variant Detection pipeline on Partek Flow platform (version 9.0.20.0819) and aligned with the mouse genome database (mm10) with the Burrow-Wheeler Aligner (BWA) (version 0.7.12). The following 3 variant callers were used: FreeBayes (version 1.0.1), Strelka (version 1.0.15), and GATK Mutect2 (version 4.0). A splenic DNA sample served as the normal control. Variants shared with spleen were considered as SNPS and removed. RNA fragments per kilobase of transcript per million mapped reads (FKPM) levels of tumor-associated antigens and neoantigens showed the extent of upregulation.

### DC2.4 TMG neoantigen plasmid nucleofection and ex vivo TIL stimulation.

DC2.4 cells were cultured for 48 hours in DC2.4 media to reach 80%–90% confluence and were then collected for nucleofection with 1 of 10 TMGs or a control GFP plasmid using the Cell Line Nucleofector Kit L (VCA-1005, Lonza). Two micrograms TMG or GFP plasmid DNA was nucleofected using the Lonza program Y-001, following the protocol for immature and mature mouse DCs. Transfection efficiency ranged between 65% and 80%. Each TMG-DC2.4 sample was resuspended in TIL media with 50 U/mL IL-2 and plated in a 96-well plate at a 10:1 ratio of TILs/DCs and cultured overnight at 37°C. The production of IFN-γ and GzmB was determined by flow cytometry or ELISA.

### ELISA.

Supernatant samples stored at –20°C and thawed on ice were then diluted 1:5 for mouse IFN-γ ELISA (430804, BioLegend) and plated in triplicate. OD_450_ and OD_570_ readings were obtained; OD_570_ values were subtracted from OD_450_, triplicate samples and averaged, and a standard curve was used to determine pg/mL concentrations. The final IFN-γ concentration was determined by multiplying the pg/mL concentration by the dilution factor.

### Antibody staining and flow cytometry.

The following surface and intracellular staining panels were used to assess surface TCR expression, the functional capacity of TILs, lymphoid/myeloid tumor distribution, and the functional response to TMG-DCs. The BD Cytofix/Cytoperm Kit with GolgiStop (BD, no. 554715) was used for intracellular staining. Samples were acquired with Cytek Aurora 3L Plate Loader and analyzed in FlowJo. For cell-surface staining and functional assays, the following BioLegend products were used: Zombie Aqua (no. 423102), BV650 CD3 (17A2, no. 100229), Alexa Fluor 700 CD4 (RM4-4, no. 116022), APC-Cy7 CD8 (53-6.7, no. 100714), PE 4-1BB (17B5, no. 106105), APC 4-1BB (17B5, no. 106110), PE-Cy7 PD-1 (29F.1A12, no. 135216), APC CD206 (C068C2, no. 141707), PE-Cy7 F4/80 (BM8, no. 123113), PerCp-Cy5.5 CD38 (90, no. 102722), BV421 GzmB (QA18A28, no. 396414), and BV711 CD107a (1D4B, no. 121614). For TIL and DC studies, the following BioLegend products were used: PerCP CD8a (53-6.7, no. 100732), PE TCRvβ6 (RR4-7, no. 140004), PE TCRvβ8.3 (1B3.3, no. 156304), PE-Cy7 IFN-γ (XMG1.2, no. 505826), BV421 GzmB (QA18A28, no. 396414), BV785 CD3 (17A2, no. 100231), APC perforin (S16009A, no. 154304), and PerCp-Cy5.5 TNF-α (MP6-XT22, no. 506322). For lymphoid/myeloid panels, the following BioLegend products were used: Zombie Aqua (no. 423102), APC/Fire-750 CD45 (30-F11, no. 103153), BV785 CD3 BV785 CD3 (17A2, no. 100231), Alexa Fluor 700 CD4 (RM4-4, no. 116022), FITC CD8α (5H10-1, no. 100803), BV605 TCR γ/δ (GL3, no. 118129), PE CD20 (SA271G2, no. 152105), APC NK1.1 (S17016D, no. 156505), APC/Fire-810 F4/80 (BM8, no. 123165), BV650 GR-1 (RB6-8C5, no. 108441), BV711 CD206 (C068C2, no. 141727), PE-Cy7 CD11b (M1/70, no. 101215), PE/Dazzle-594 CD11c (N418, no. 117347), and PerCP I-A/I-E (M5/114.15.2, no. 107623). Also for lymphoid/myeloid panels, the following BD Biosciences products were used: BV480 TCRvβ9 (MR10-2, no. 746449), BV480 TCRvβ10[b] (B21.5, no. 746729), BV650 TCRvβ11 (RR3-15, no. 743679), BV650 TCRvβ13 (MR12-3, no. 743993), BV711 TCRvβ2 (B20.6, no. 745428), BV711 TCRvβ3 (KJ25, no. 743416), BV785 TCRvβ5.1,5.2 (MR9-4, no. 743003), BV785 TCRvβ 8.1,8.2 (MR5-2, no. 744334), FITC TCRvβ7 (TR310, no. 553215), FITC TCRvβ14 (14-2, no. 553258), BV480 CD45.1 (A20, no. 746666), and the anti-Vβ FITC TCR kit (no. 557004). Detailed reagents, panels, procedures, and the FlowJo analysis strategy are provided in the Supplemental Methods.

### Study approval.

All mice were housed at and all animal procedures were approved by the IACUC of the University of Colorado Anschutz Medical Campus.

### Statistics.

NanoString data were analyzed with nSolver, and Rosalind software and statistically significant changes were identified as *P* values of less than 0.05 and fold changes of greater than ±1.5. Remaining analysis was completed with GraphPad Prism (GraphPad Software). Differences between treatment groups were determined by 2-way ANOVA. Differences between TCRvβ groups were determined by multiple unpaired, 2-tailed *t* tests relative to the untreated control. Differences between patient groups from TCGA data were determined by unpaired Mann-Whitney *U* test.

### Data availability.

Values for all data points in graphs are reported in the Supporting Data Values file. The data generated in this study are available upon request from the corresponding author. The publicly available TCGA datasets from patients with melanoma were directly downloaded from TCGA Data Portal (https://tcga-data.nci.nih.gov/tcga/).

## Author contributions

ED, AJN, and GKA designed the research studies. AJN, GKA, AS, JC, JL, ASD, DG, and XB conducted experiments and acquired and analyzed data. ED, AJN, GKA, and BAW wrote and/or edited the manuscript. WR, MM, RT, AM, KC, provided reagents and patient samples. ED, GKA, JDH, CA, and KC acquired and analyzed genetic data. AJN is listed as the first co-author because she performed the majority of the experiments.

## Supplementary Material

Supplemental data

Unedited blot and gel images

Supporting data values

## Version 1 10/22/2024

In-Press Preview

## Version 2 12/16/2024

Electronic publication
