# Supplementary material for: DNA-PK inhibition enhances neoantigen diversity and increases T cell responses to immunoresistant tumors
Source: J Clin Invest. 2024 Oct 22;134(24):e180278. doi: 10.1172/JCI180278 (PMC11645140; doi:10.1172/JCI180278)
Supplement: Supplemental data [file jci-134-180278-s169.pdf]

NU-SL40/No Drug

Gene	Log2(FoldChange)	Gene	Log2(FoldChange)	Gene	Log2(FoldChange)
CXCL9	3.974	ISG15	1.664	IRF9	0.911
CCL5	3.375	TAPBP	1.635	HK2	0.908
GBP2	3.275	*NOS2	1.570	RSAD2	0.894
H2-AA	3.228	TRIM21	1.567	ICAM2	0.887
CD74	3.150	LILRB4A	1.552	CD36	0.840
H2-EB1	3.105	CXCL10	1.550	MRC1	0.833
H2-T23	3.046	MMP12	1.524	SGK1	0.819
PSMB8	2.867	C1QB	1.501	^NFIL3	0.805
PSMB9	2.799	STAT2	1.486	IFI27	0.779
H2-AB1	2.724	GIMAP4	1.467	EIF2AK2	0.627
GBP3	2.577	CCL7	1.423	STAT3	0.595
CXCL16	2.544	B2M	1.419	PFKFB3	0.553
IL18BP	2.536	CCL12	1.362	C2	0.542
AXL	2.529	THBS1	1.329	NFKBIA	0.534
LY6C1	2.442	CCL2	1.324	IRF2	0.517
TAP2	2.441	PARP9	1.309	DAB2	0.505
IFIT3	2.405	IFIH1	1.257	IFIT2	0.496
IFI203	2.279	CCL9	1.243	CD47	0.480
TAP1	2.254	IFI35	1.217	BCL2L1	0.462
CD274	2.197	PARP12	1.216	TGFBR2	0.443
CCL8	2.178	DTX3L	1.214	PPARGC1B	0.382
H2-DMB1	2.171	ATF3	1.189	HDAC3	-0.146
FCGR4	2.168	HERC6	1.179	ITGA4	-0.298
SLC11A1	2.084	IFIT1	1.175	^CXCR3	-0.345
CTSS	2.081	CD68	1.136	TLR4	-0.361
IRF1	1.999	SPP1	1.106	MYC	-0.418
PSMB10	1.927	FCGR2B	1.089	#TNFRSF10B	-0.453
NLRC5	1.913	CEBPB	1.067	ADAM12	-0.674
LYZ1	1.904	SBNO2	1.058	CD276	-0.787
H2-K1	1.850	OAS1A	1.044	SOX11	-0.811
C1QA	1.804	THY1	1.040	TGFB2	-0.991
H2-D1	1.768	ITGB3	0.950	GLS	-1.072
ADGRE1	1.743	LGALS9	0.923	*TYMS	-1.411

NU/No Drug

Gene	Log2(FoldChange)
*NOS2	1.399
SAMSN1	1.051
THBS1	0.983
PMS2	0.786
^NFIL3	0.698
*TYMS	-1.068
^CXCR3	-1.322

SL40/No Drug

Gene	Log2(FoldChange)
*NOS2	1.365
SBNO2	0.680
#TNFRSF10B	-0.595
CDKN1A	-0.789
*TYMS	-0.845
JAG1	-0.868
TNFRSF11B	-0.948

Color scale: Log2(FoldChange), from -1.5 to 4.0

Supplemental Table 1 – Nanostring Pan Cancer IO differentially regulated gene expression. List of differentially regulated genes determined by Nanostring Pan Cancer IO Gene Expression Analysis. Only genes that were statistically significant (p-value < 0.05, fold-change more than +/- 1.5) were included. (*) Indicates gene differentially regulated in all treatments. (^) indicates gene differentially regulated in NU-SL40 and NU. (#) Indicates gene differentially regulated in NU-SL40 and SL40.

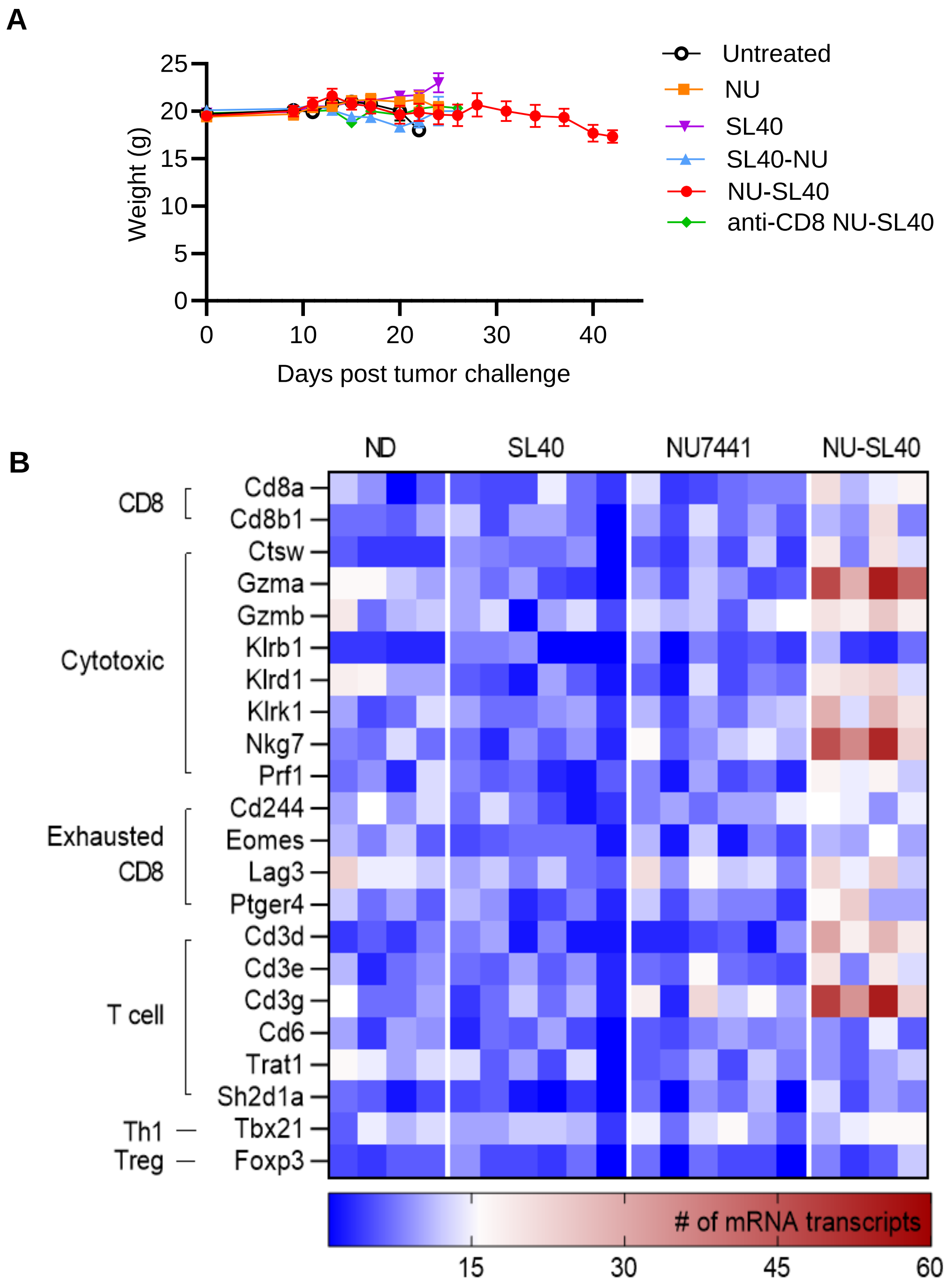

Supplemental Figure. 1 A) C57BL/6 mice were injected with B16-F10 tumor cells, and once tumors were established (25mm^2), mice were either left untreated or received NU alone, SL40 alone, combination SL40-NU, or combination NU-SL40. CD8 T cells were depleted by administration of anti-CD8 prior to treatment with NU-SL40. CD8 depletion was administered twice prior to NU-SL40 treatment. Weight represents group means +/- SEM – untreated, n=10; NU (n=10); SL40 (n=10); SL40-NU (n=11); NU-SL40 (n=8); CD8 depleted NU-SL40 (n=10). **B)** Heatmap of T cell associated genes by number of mRNA transcripts extracted from whole B16 tumors generated using Nanostring Pan Cancer IO gene expression panel.

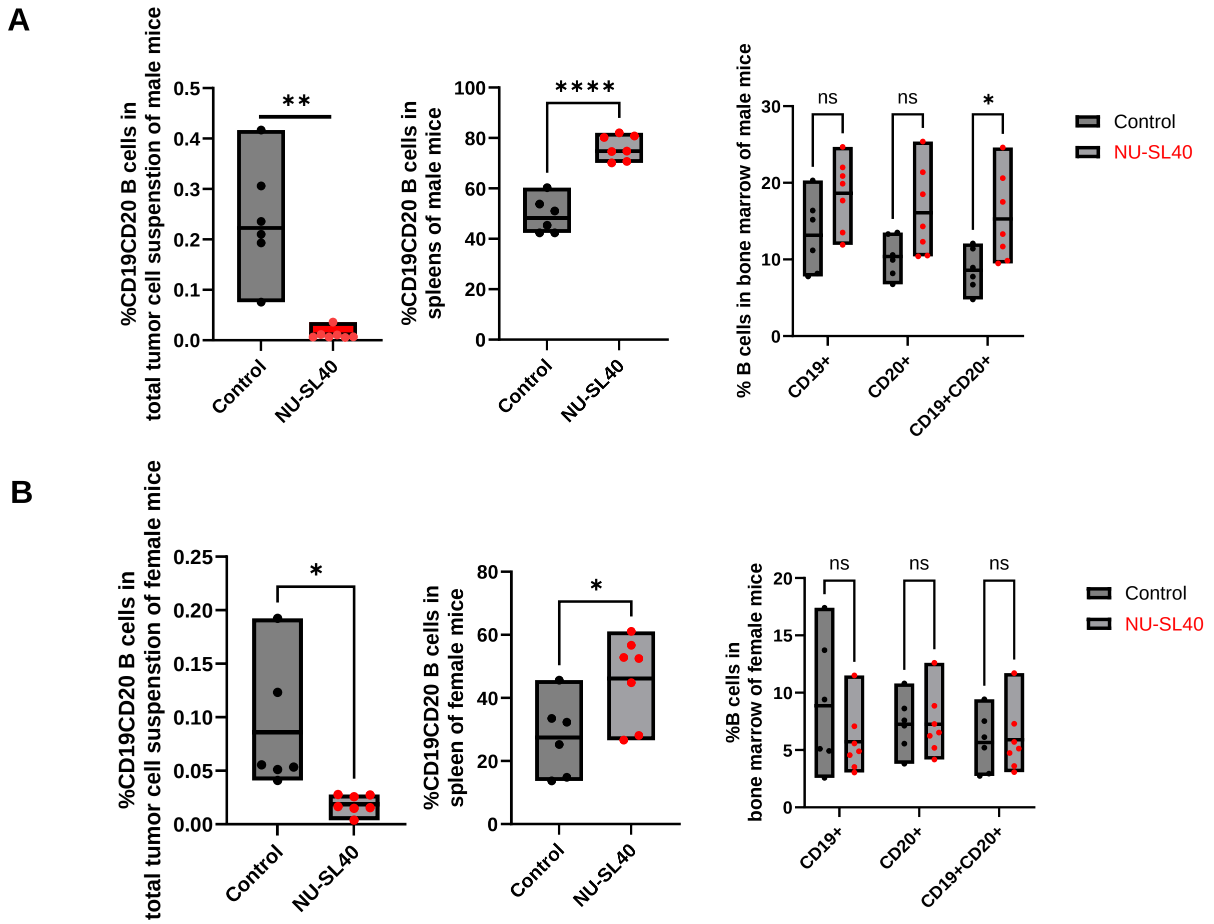

Supplemental Figure 2. B16-F10 tumor-bearing male or female mice were treated with or without combination NU-SL40 and 10 days after treatment, tissues were collected to analyze B cell populations by flow cytometry. Frequency of B cell populations in the tumor (left), spleen (middle), and bone marrow (right) in **A)** male and **B)** female mice. Untreated (n=6); NU-SL40 treated (n=7). Statistical tests were performed using student's *t* tests. *$P < 0.05$, **$P < 0.01$, ****$P < 0.0001$.

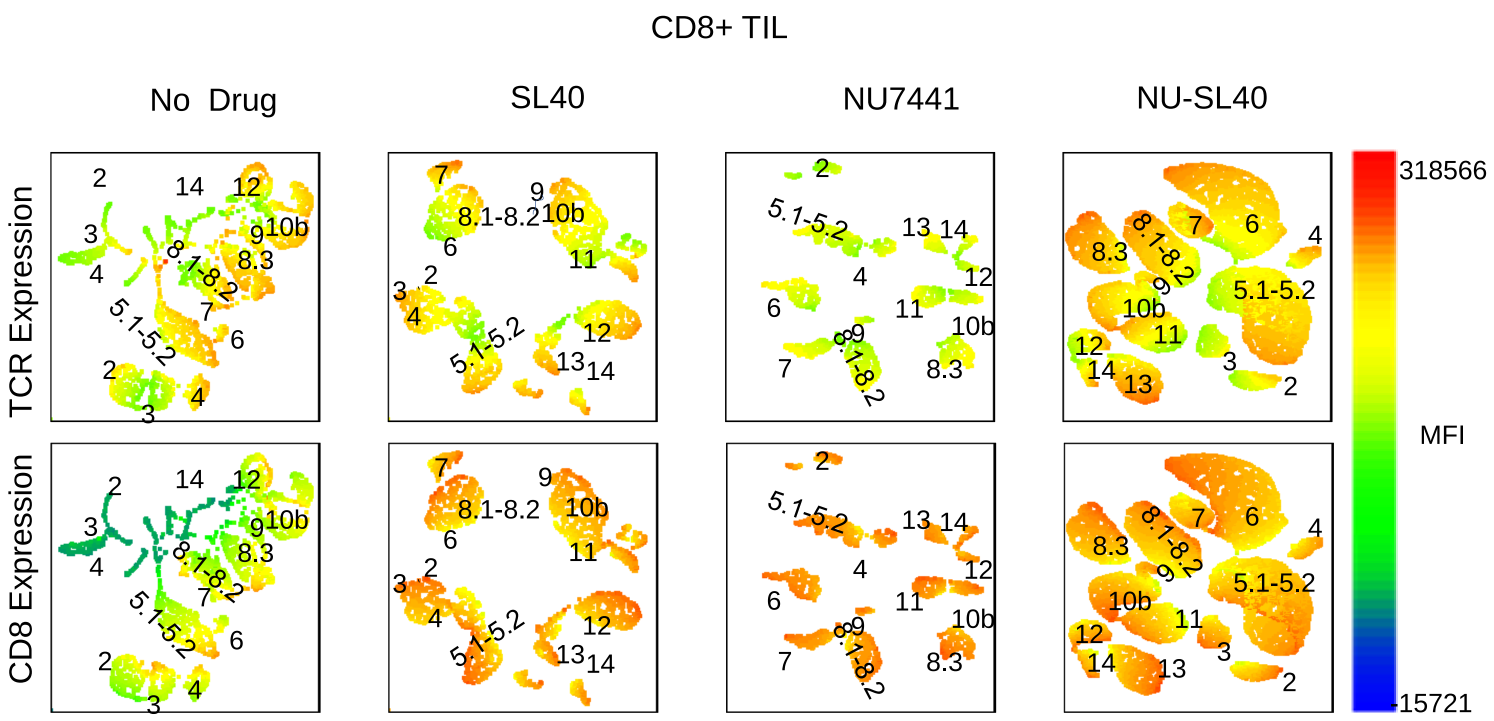

Supplemental Figure 3. C57BL/6 mice were injected with B16-F10 tumor cells, and once tumors were established (25mm^2), mice were either left untreated or received NU alone, SL40 alone, or combination NU-SL40. Ten days after mice received SL40, whole tumor samples were stained with antibodies against CD3, CD8, CD45, and the indicated TCRVβ. Plots show the raw distribution CD8 TCRVβ family members with relative TCR or CD8 protein density levels on the surface of specific CD45+CD3+CD8 TILs. Untreated (n=15); SL40 (n=9); NU (n=5); NU-SL40 (n=8).

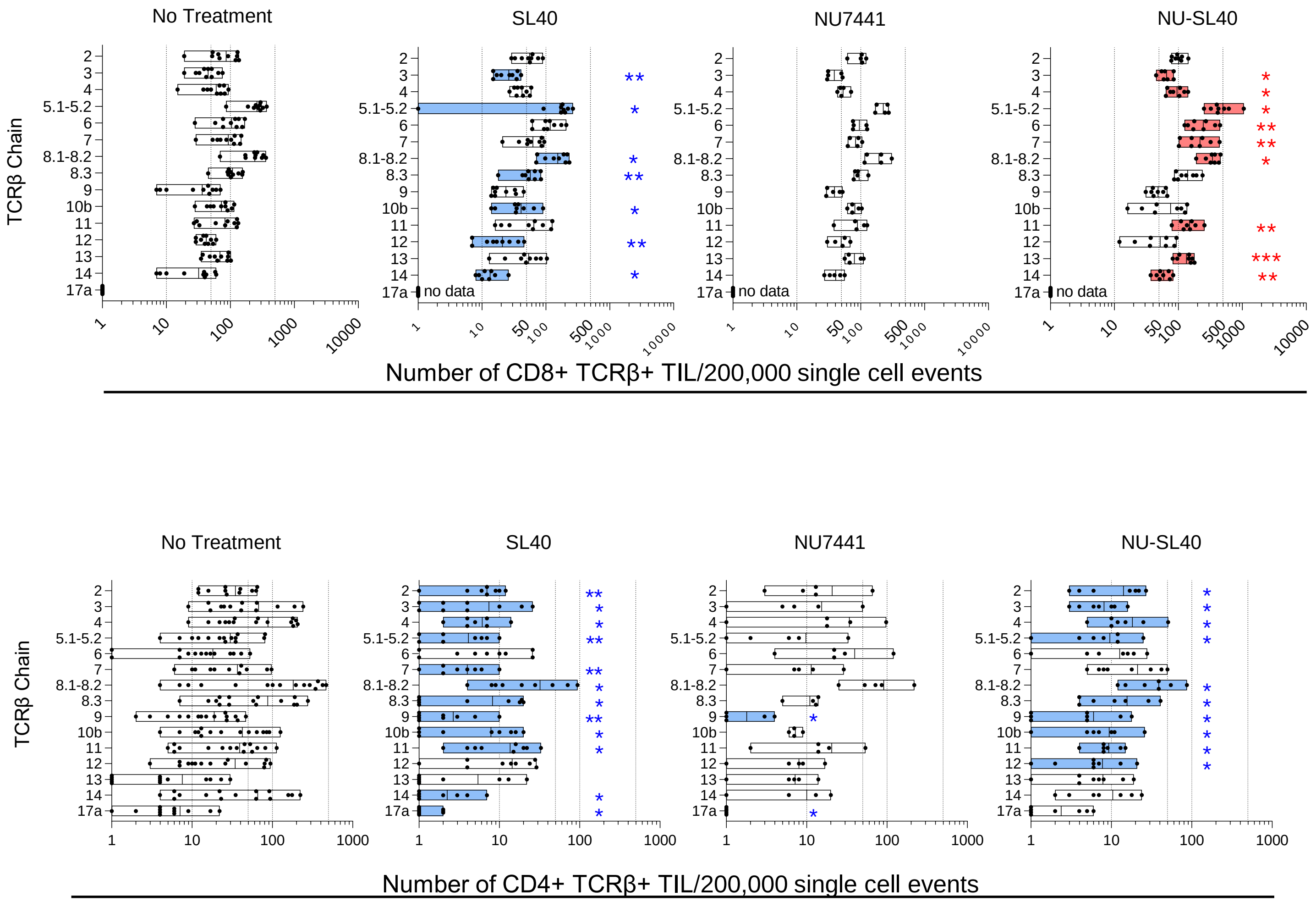

Supplemental Figure 4. CD8 and CD4 TCRvβ repertoire distribution in the circulation of tumor-bearing mice. B16-F10 tumor-bearing mice were treated as described in Figure 1A and blood collected between 10 and 12 days post immune adjuvant vaccine. After red blood cell lysis, cells were stained with antibodies against CD3, CD4, and TCR and analyzed by flow cytometry. The number of TCRvβ chain per 200,000 single cell events is shown. Blue and red bars represent significant decreases or increases, respectively, in TCRvβ counts in treatment conditions compared to no treatment. Each dot represents data collected from one mouse. Untreated (n=5); SL40 (n=5); NU (n=5); NU-SL40 (n=4). Statistical significance determined by multiple unpaired t-tests. * $p<0.05$, ** $p<0.01$, *** $p<0.001$, **** $p<0.0001$.

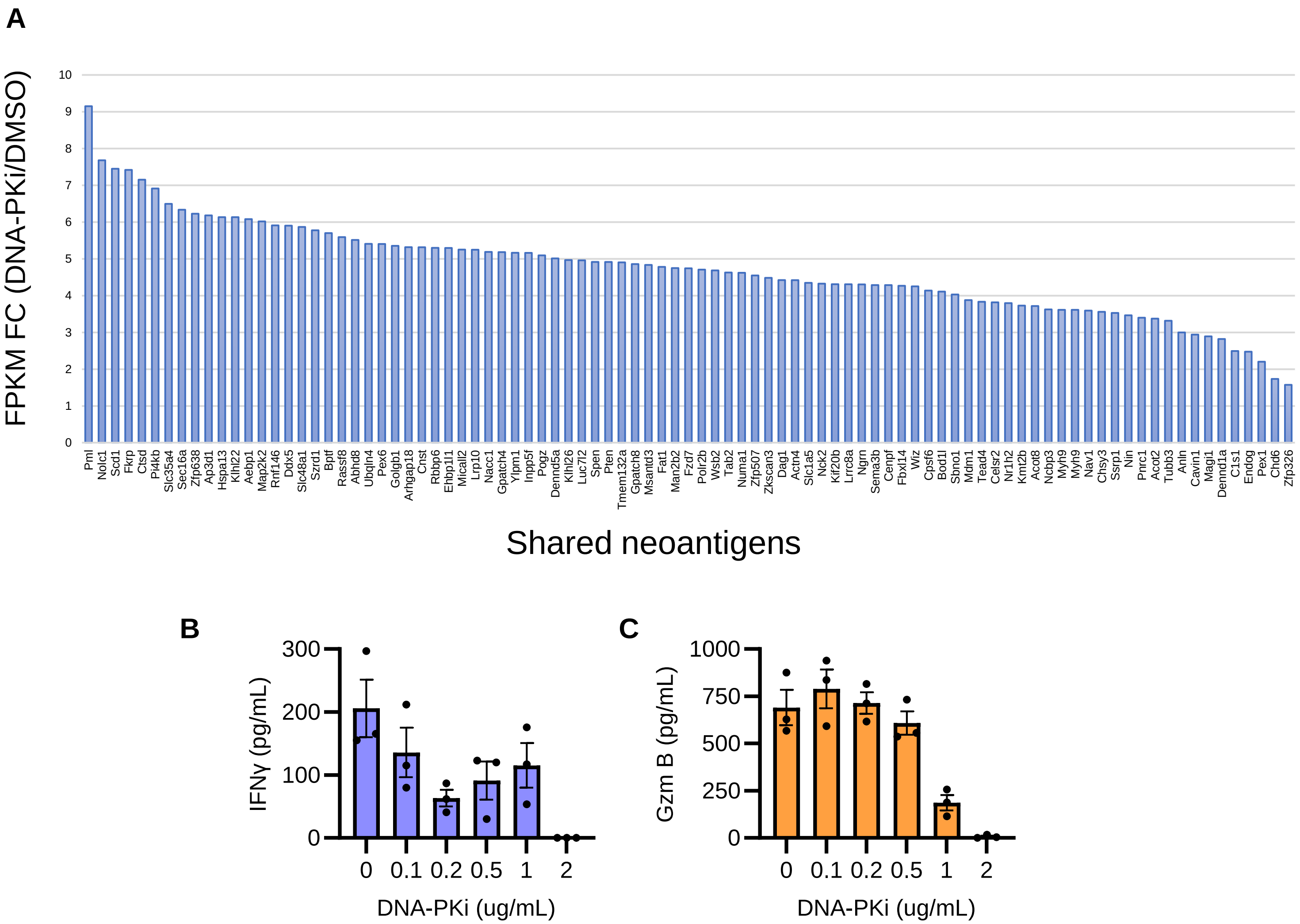

Supplemental Figure 5. A) B16-F10 melanoma cells were treated with DMSO and 2µM NU7441 for 72 hours and neoantigens identified as described previously (Lu et al. *Clin Cancer Res.* 2014;20(13):3401-10). The bar graph represents the FPKM levels of neoantigen-producing genes shared between DMSO and 2µM NU7441. Polyclonal TIL from human melanoma patient donor were activated with anti-CD3/-CD28 beads (1:20) with titrated concentrations of NU7441 (ug/mL) for 96hrs. Bar graphs indicate **B)** interferon gamma (1:800 dilution) and **C)** granzyme B (1:200 dilution) production by ELISA of triplicate samples (pg/mL).

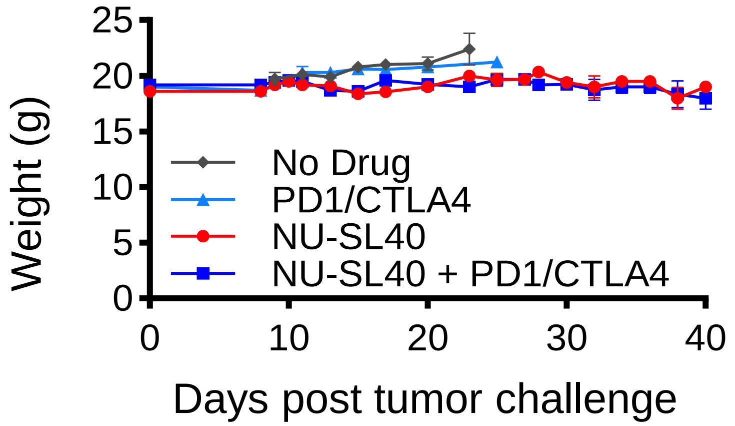

Supplemental Figure 6. C57BL/6 mice with established (~25mm^2) B16-F10 tumors remained untreated, treated with PD-1/CTLA-4 blockade, NU-SL40, or NU-SL40 in conjunction with PD-1/CTLA-4 blockade as described in the materials and methods section. Mouse weight (grams) was monitored over time.

A

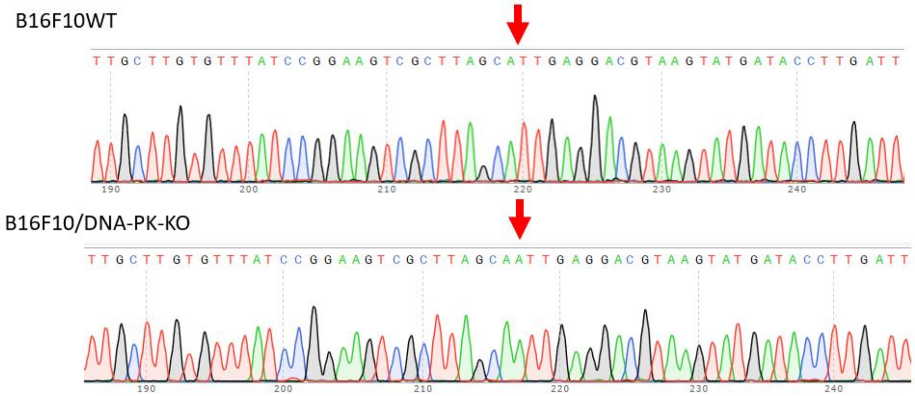

B

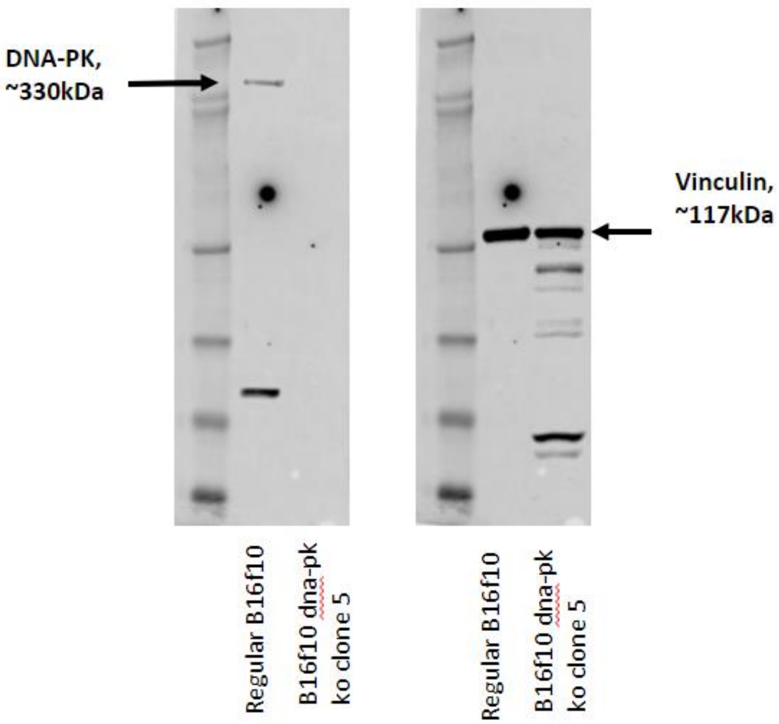

Supplemental Figure 7. DNA-PK was knocked out in B16-F10 melanoma cells using CRISPER/Cas9 vectors targeting exon #3. **A)** Genomic sequencing data confirming mutation in the indicated nucleotide (red arrow). **B)** Western blot analyses to confirm the knocking out of DNA-PK in B16-F10 (left panel) and equal protein loading (right panel).
