# Supplementary material for: DNA-PK inhibition enhances neoantigen diversity and increases T cell responses to immunoresistant tumors
Source: J Clin Invest. 2024 Oct 22;134(24):e180278. doi: 10.1172/JCI180278 (PMC11645140; doi:10.1172/JCI180278)

Figure 4D: “Tyrp-1” and “a-tubulin”

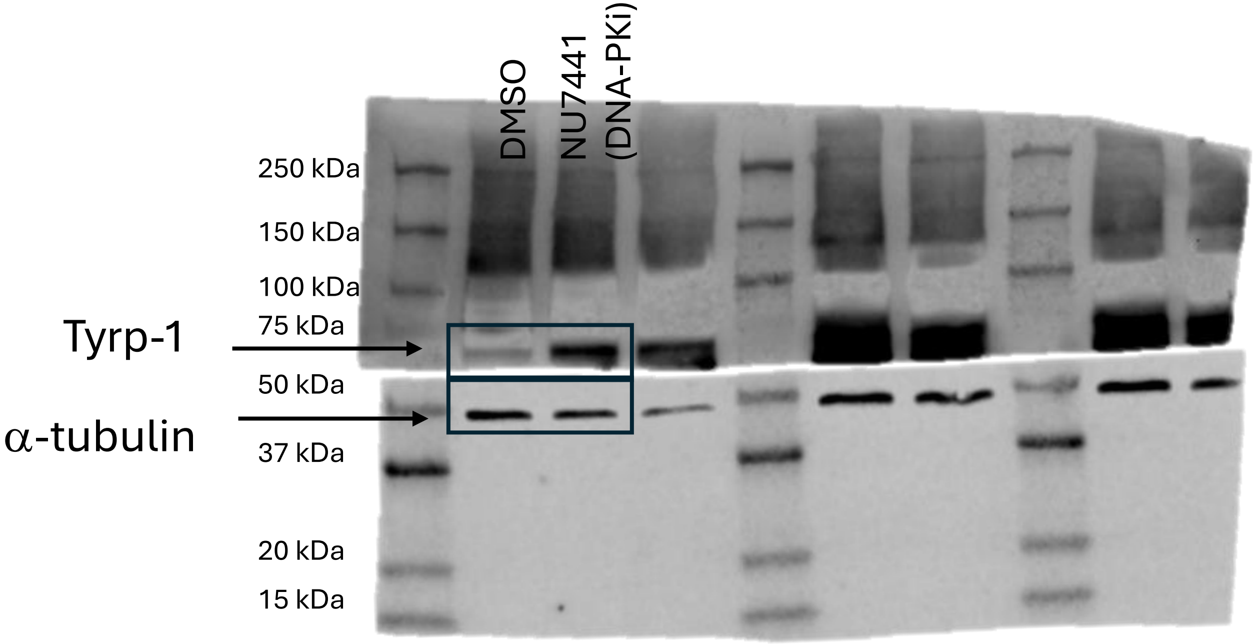

This membrane started as a single membrane but was cut so that the membrane could fit in our staining chamber. This allowed us at the time to use less antibody. This also allows us to use the lower half membrane for other stains if needed.

Figure 4D “Gp100”

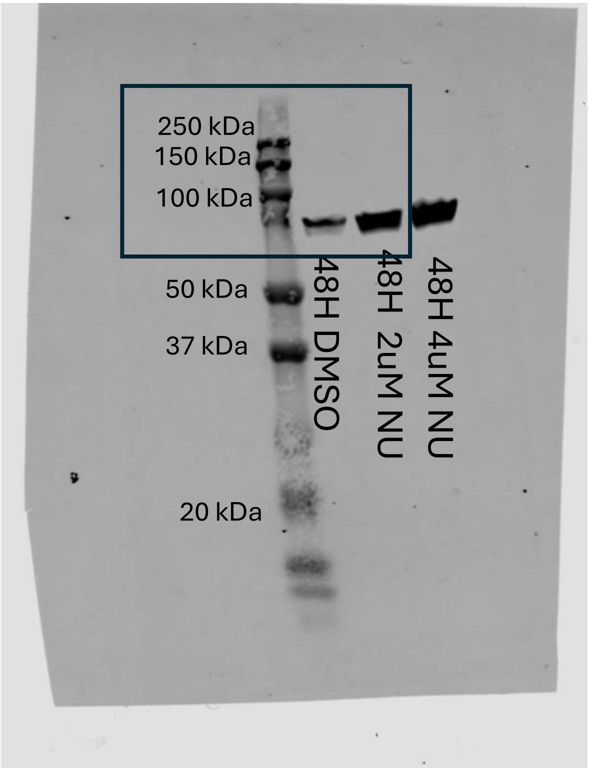

Figure 4D “B-actin”

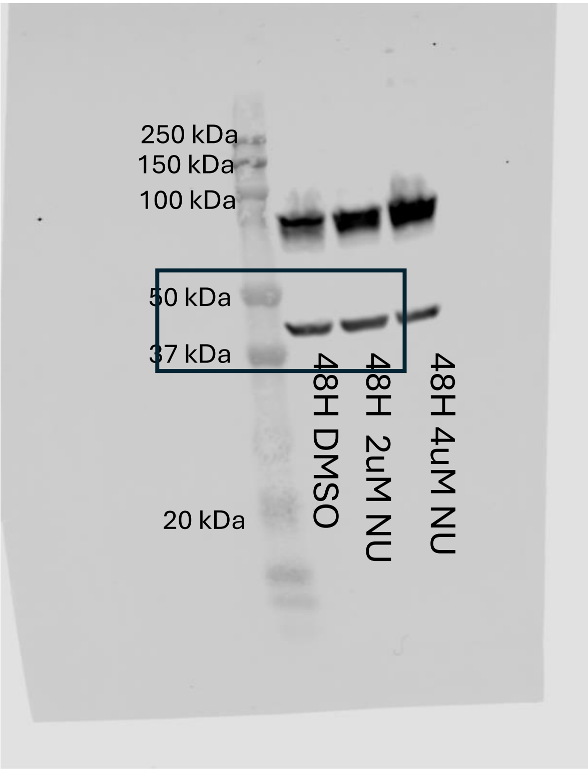

Figure 4I “Mart-1”

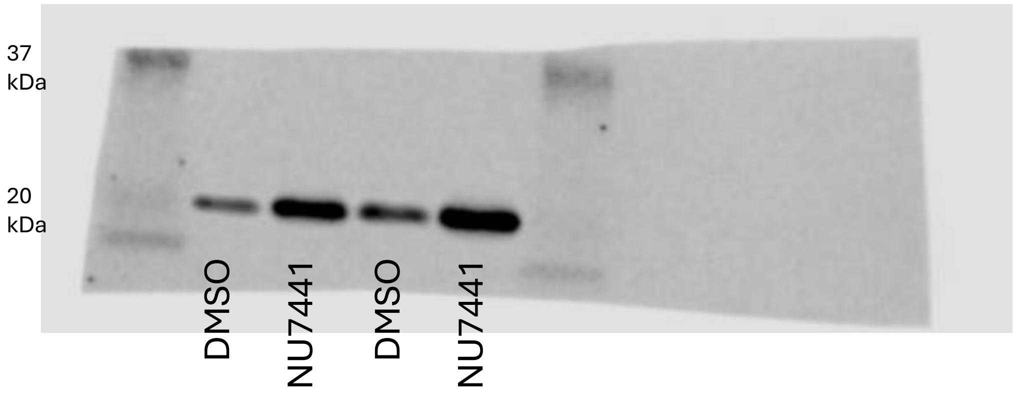

Figure 4I “a-tubulin”

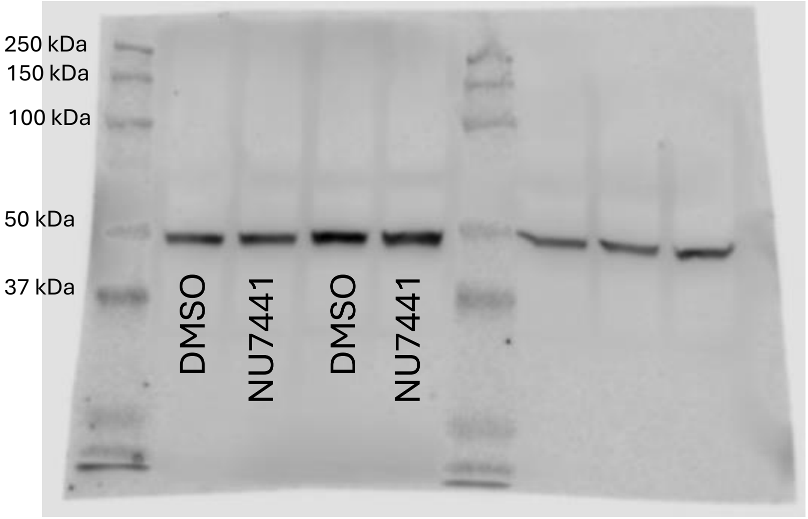

Figure 4I: "Tyrp-1"

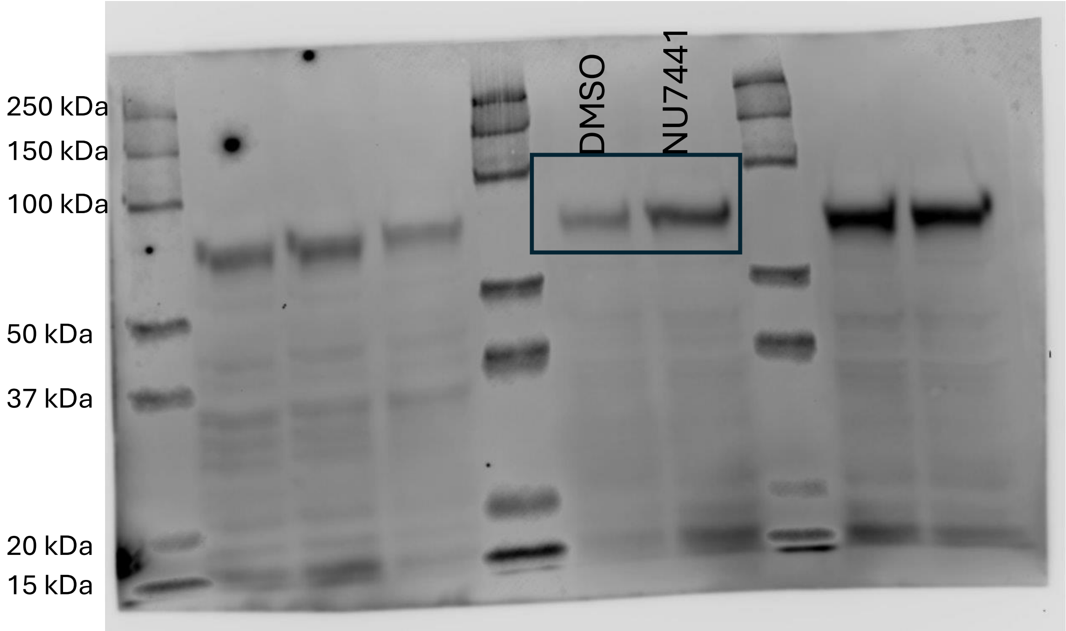

Figure 4I: "b-actin"

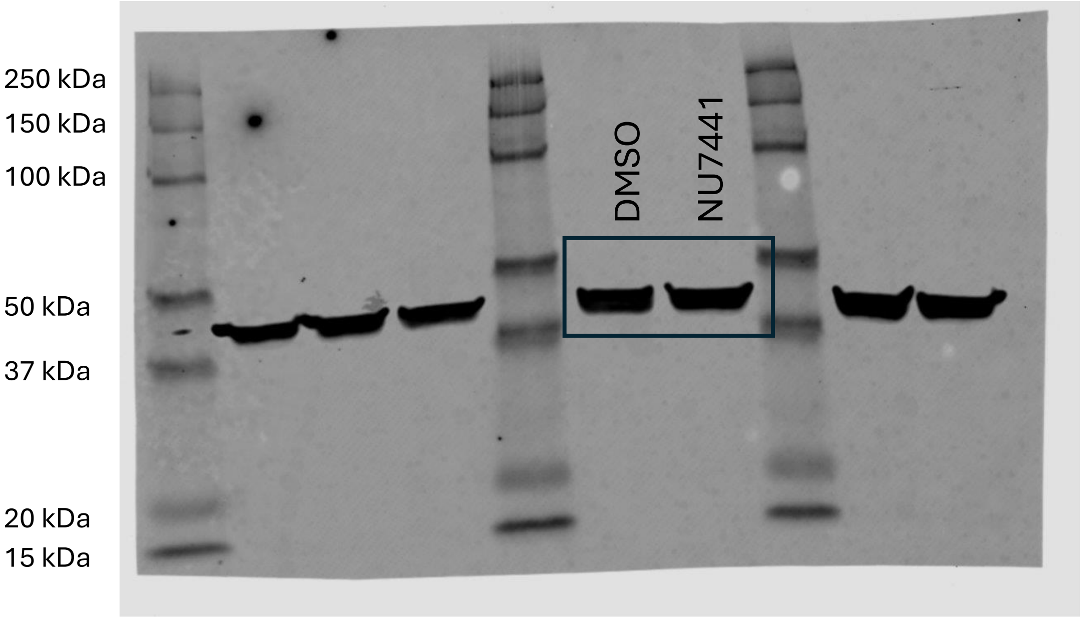

Figure 4I: “gp100”

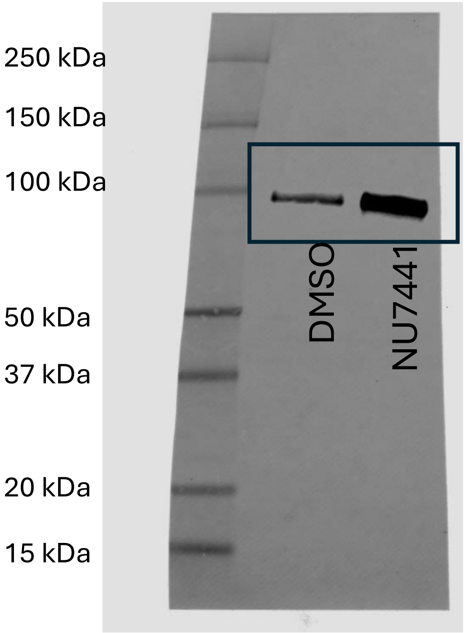

Figure 4I: “b-actin”

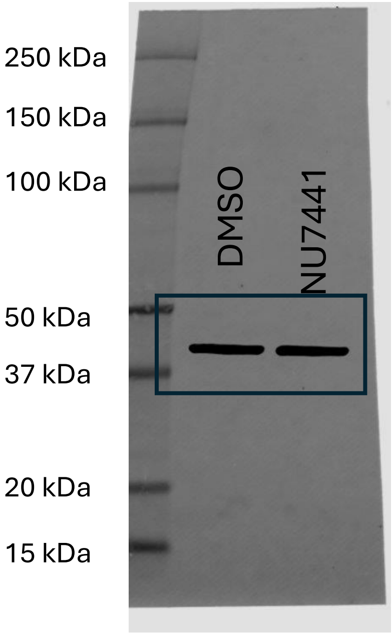

Supplement: Unedited blot and gel images [file jci-134-180278-s170.pdf]
